# Utility of Routine Laboratory Biomarkers to Detect COVID-19: A Systematic Review and Meta-Analysis

**DOI:** 10.3390/v13050803

**Published:** 2021-04-30

**Authors:** Jana Suklan, James Cheaveau, Sarah Hill, Samuel G. Urwin, Kile Green, Amanda Winter, Timothy Hicks, Anna E. Boath, Ashleigh Kernohan, D. Ashley Price, A. Joy Allen, Eoin Moloney, Sara Graziadio

**Affiliations:** 1 NIHR Newcastle In Vitro Diagnostics Co-operative, Translational and Clinical Research Institute, William Leech Building, Medical School, Newcastle University, Newcastle NE2 4HH, UK; kile.green@newcastle.ac.uk (K.G.); david.price15@nhs.net (D.A.P.); joy.allen@newcastle.ac.uk (A.J.A.); 2 Department of Infectious Diseases, Royal Victoria Infirmary, The Newcastle Upon Tyne Hospitals NHS Foundation Trust, Newcastle upon Tyne NE1 4LP, UK; james.cheaveau1@nhs.net; 3 Health Economics Group, Population Health Sciences Institute, Faculty of Medical Sciences, Newcastle University, Baddiley-Clark Building, Newcastle University, Newcastle upon Tyne NE2 4AX, UK; Sarah.Hill2@newcastle.ac.uk (S.H.); A.E.Boath2@newcastle.ac.uk (A.E.B.); ashleigh.kernohan@newcastle.ac.uk (A.K.); eoin.moloney@optimaxaccess.com (E.M.); 4 NIHR Newcastle In Vitro Diagnostics Co-operative, The Newcastle Upon Tyne Hospitals NHS Foundation Trust, William Leech Building, Medical School, Newcastle upon Tyne NE2 4HH, UK; sam.urwin@newcastle.ac.uk (S.G.U.); amanda.winter@newcastle.ac.uk (A.W.); timothy.hicks@nhs.net (T.H.); sara.graziadio@york.ac.uk (S.G.)

**Keywords:** systematic review, meta-analysis, COVID-19, diagnosis, biomarker

## Abstract

No routine laboratory biomarkers perform well enough in diagnosing COVID-19 in isolation for them to be used as a standalone diagnostic test or to help clinicians prioritize patients for treatment. Instead, other diagnostic tests are needed. The aim of this work was to statistically summarise routine laboratory biomarker measurements in COVID-19-positive and -negative patients to inform future work. A systematic literature review and meta-analysis were performed. The search included names of commonly used, routine laboratory tests in the UK NHS, and focused on research papers reporting laboratory results of patients diagnosed with COVID-19. A random effects meta-analysis of the standardized mean difference between COVID-19-positive and -negative groups was conducted for each biomarker. When comparing reported laboratory biomarker results, we identified decreased white blood cell, neutrophil, lymphocyte, eosinophil, and platelet counts; while lactate dehydrogenase, aspartate aminotransferase, and alanine aminotransferase were elevated in COVID-19-positive compared to COVID-19-negative patients. Differences were identified across a number of routine laboratory biomarkers between COVID-19-positive and -negative patients. Further research is required to identify whether routine laboratory biomarkers can be used in the development of a clinical scoring system to aid with triage of patients.

## 1. Introduction

Severe acute respiratory syndrome coronavirus 2 (SARS-CoV-2) has rapidly spread throughout the world, with significant morbidity and mortality occurring alongside global disruption [1]. The number of cases worldwide continues to increase and is likely to continue to rise further without interventions such as effective contact tracing, social distancing, lockdowns, or vaccination. Common symptoms of the disease include fever, cough, sore throat, headache, fatigue, myalgia, breathlessness, anosmia, and ageusia [2]. 

The laboratory identification of patients with COVID-19 has been challenging. Furthermore, many countries are currently experiencing a second or third peak of infections [3], placing significant stress on testing capacity in many locations. The main test used to support diagnosis is reverse transcription polymerase chain reaction (RT-PCR) [4] on nasopharyngeal samples. Limitations of RT-PCR are the relatively long turn-around time as well as imperfect sensitivity [5]. 

Routine laboratory biomarkers can provide an overall picture of the health status of a patient in acute medical settings. However, no routine laboratory biomarkers perform well enough in isolation for diagnosing COVID-19 for them to be used as a standalone diagnostic test or to help clinicians prioritize patients for treatment [6]. There have been some attempts in combining several biomarkers and other parameters into a clinical scoring algorithm to aid COVID-19 diagnosis [7], but these models are often poorly reported, at high risk of bias due to poor reporting, poor methodological conduct, and lacking robust validation. Using these models in practice may result in performance characteristics that are lower than those reported in the literature [8]. 

The objective of this study is to conduct a systematic review and meta-analysis of routine laboratory biomarkers that are readily accessible across Emergency Departments in the UK NHS and summarise routine laboratory biomarker measurements in COVID-19-positive and -negative patients. This work could help inform further development of clinical scoring algorithms, which are likely to be important for use in clinical settings that do not readily have access to point-of-care (POC) or laboratory testing for COVID-19.

## 2. Materials and Methods

### 2.1. Search Strategy and Selection Criteria

The OVID Medline database, Living Systematic review project [9] and the Living Overview of the Evidence (L OVE) Platform [10] were electronically searched on the 23 September 2020 by JS to identify primary studies including laboratory test results for COVID-19-positive and -negative patients. A search strategy was developed in consultation with an Information Specialist. The full search criteria are presented in Appendix A. No language or country restrictions were applied. Date was restricted from December 2019. Systematic review and meta-analysis studies were examined to identify additional primary studies. Articles were also identified through snowballing procedures, by other authors, searching the grey literature, and through use of Google Scholar. Records were imported into EndNote^®^ X9 (Thomas Reuters, Toronto, ON, Canada).

### 2.2. Inclusion and Exclusion Criteria

The inclusion criteria were as follows:

1. Published or un-published (pre-print) primary studies or secondary studies where data were used to inform a mathematical model;
2. The population included patients suspected of having COVID-19 in hospitals based on their signs and symptoms and where the diagnosis of COVID-19 was confirmed with RT-PCR testing, and radiology;
3. Studies reporting the mean (with or without standard deviation) or studies reporting median (with uncertainty measures) of biomarker levels under investigation.

The exclusion criteria were:

1. Analysis of population-based studies with only secondary outcomes such as fatality rate, without discussing the primary outcomes;
2. Analysis carried out exclusively on specific sub-populations (elderly, pregnant women, children);
3. Case reports and studies with a sample size of less than 10;
4. Biomarkers with less than 5 studies were excluded from the meta-analysis;
5. Studies reporting biomarker levels, but exclusively for prognostic research or in correlation with severity of disease
6. Studies that compare COVID-19-positive patients with controls and patients that have not been tested using RT-PCR (e.g., comparison with other pandemic or with previous flu seasons);
7. Studies reporting only the number of patients outside the normal ranges.

### 2.3. Patient Population

COVID-19-positive cases included patients highly suspected to have the disease as determined by a positive RT-PCR test result, imaging, or other diagnostic criteria where applicable. COVID-19-negative refers to patients with a negative RT-PCR test result two or more times in a row, or clinically treated as negative for COVID-19 based on clinical criteria (usually including imaging). The setting was secondary care (hospitals).

### 2.4. Study Selection 

After removing duplicates, titles, abstracts, and full texts were screened by two independent reviewers (JS, EM), with any disagreements resolved by a third reviewer (SG) (blinded to the original decisions). Records that were not published in English were translated to English using Google Translate. 

### 2.5. Data Extraction and Quality Assessment

Two reviewers (JS, SH) extracted relevant data independently in three different spreadsheets: (1) the table of characteristics; (2) the pre-designed extraction form to report biomarker average levels for COVID-19-positive and -negative patients (Table S1); (3) the risk of bias table (see Table S2). 

The data collection forms were designed by JS and SG and piloted on 2 articles by other reviewers (AW and KG) before the data extraction started. For each biomarker and for each group (positive and negative for COVID-19), a pair of two independent reviewers (JS, SH, AW, KG, SGU, TH, AEB and AK) extracted the mean, median, standard deviation, range, and interquartile range. 

We adapted the QUADAS-2 [11] tool to assess the quality of the selected articles. QUADAS-2 is designed to assess risk of bias in diagnostic accuracy studies, but the primary aim of this review was a comparison of means; therefore, we excluded the comparison between the index and reference test pillar. The modified risk of bias tool included 11 quality assessment items within three QUADAS-2 domains covering the patient selection, testing, flow, and timing. The full list of questions about the risk of bias that were considered is presented in Table S2. Studies that were considered prone to bias in six or more areas were classified as being at high risk of bias. This threshold was chosen arbitrarily by the authors. 

### 2.6. Data Synthesis and Statistical Analysis

The pooled standardised mean difference (SMD) with 95% confidence interval (CI) was calculated for each biomarker and each group of patients (COVID-19-positive and -negative). When the study reported median (range or interquartile range—IQR), mean and standard deviation were estimated using the formula proposed by Hozo et al. [12]. When the standard deviation or standard error were not reported, we imputed the missing value using the average standard deviation pooled from other studies [13]. When data were reported separately for multiple groups of patients (i.e., male and female) we grouped them into COVID-19-positive and -negative patients. 

**Figure A1:**
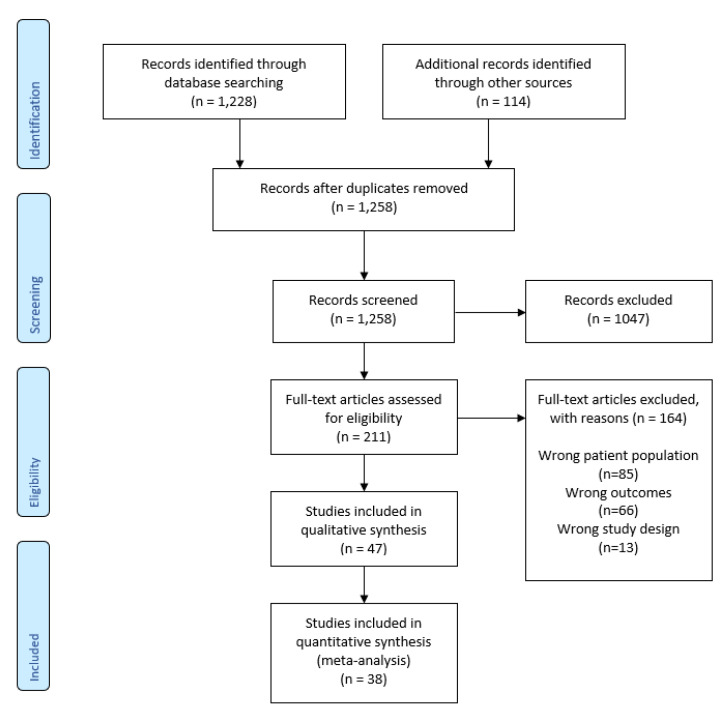
PRISMA flow diagram.

Heterogeneity was evaluated using I^2^ statistics, where a heterogeneity greater than 75% was considered high [14]. We used a random-effect model for the meta-analysis because of the high heterogeneity of the studies. Publication bias was visualized with funnel plots. The online version of Cochrane’s Review Manager [15] was used for the meta-analysis. Sensitivity analysis was carried out to focus on low risk of bias studies and published studies. All statistical tests were interpreted using the significance threshold *p* < 0.05. The Preferred Reporting Items for Systematic Reviews and Meta-analyses (PRISMA) guidelines for reporting [16] were followed (see Table S3). A PRISMA flow diagram is reported in Appendix B (see Figure A1).

## 3. Results

### 3.1. Literature Retrieval

The search identified 1258 articles. After duplicate removal, title, abstract and full-text screening, 38 studies were included in this systematic review and meta-analysis.

### 3.2. Characteristics of Included Studies

Sixty-six per cent of the studies were conducted in China. The majority were published articles, but 21% were still in pre-print on 27 January 2021. Articles were either published or submitted between 23 February 2020 and 18 September 2020, and all reported data were collected between 20 December 2019 and 27 June 2020. The average sample size was 346 patients (SD = 613.6), of which 42% of patients on average were considered COVID-19-positive (SD = 18.7%) (see Table S4); however, studies used different criteria to define COVID-19-positive patients. The COVID-19 diagnoses were based on the official guidelines available at the time of testing in the country where the data collection occurred. Generally, RT-PCR was used as the reference standard for COVID-19 as opposed to a composite reference standard. 

### 3.3. Quality Assessment

Twenty-six out of 38 studies were considered at low risk of bias (Table S2). Funnel plots are all distributed relatively symmetrically showing the absence of publication bias in the analysis (see Table S5). Additional factors which may have introduced bias were (1) a high proportion of studies reporting data from Chinese hospitals from an early point in the pandemic, which may have limited applicability to UK NHS hospitals, (2) the diagnostic criteria changed during the course of the pandemic as knowledge of the disease increased, and (3) papers that were still in pre-print long after the submission.

### 3.4. Meta-Analysis

The search strategy included selected biomarkers (see Table S1) that are readily accessible across Emergency Departments in the UK NHS. Not all of the included studies reported data for all of the biomarkers. 

Laboratory test results of confirmed COVID-19 cases were within the normal reference ranges set by laboratories across the NHS UK. Biomarkers that were significantly lower in COVID-19-positive patients than in COVID-19-negative patients were: white blood cell (SMD = −0.68, 95% CI (−0.98, −0.38), *p* < 0.001), neutrophil (SMD = −0.49, 95% CI (−0.67, −0.31), *p* < 0.001), lymphocyte (SMD= −0.47, 95% CI (−0.63, −0.31), *p* < 0.001), eosinophil (SMD = −0.85, 95% CI (−1.3, −0.4), *p* < 0.001) and platelet count (SMD = −0.47, 95% CI (−0.66, −0.28), *p* < 0.001). Biomarkers that were significantly higher in COVID-19-positive patients than in COVID-19-negative patients were: lactate dehydrogenase (SMD = 0.42, 95% CI (0.02, 0.83), *p* = 0.04), aspartate aminotransferase (SMD = 0.45, 95% CI (0.26, 0.64), *p* < 0.001) and alanine aminotransferase (SMD = 0.49, 95% CI (0.25, 0.72), *p* < 0.001). The studies included in the review showed high heterogeneity (>85% I^2^ statistic) (see Table S6).

### 3.5. Sensitivity Analysis

We performed two sensitivity analyses. One that included only studies at low risk of bias and another that included only published studies. When we removed the studies at high risk of bias (12 out of 38 studies), the level of heterogeneity decreased but remained high across all studies (>60% I^2^ statistic) (see Table S7). The results of the sensitivity analysis remained stable across white blood cell, neutrophil, lymphocyte, eosinophil, and platelet count biomarkers. The results indicate that these values were still significantly lower in COVID-19-positive patients compared to COVID-19-negative patients. For lactate dehydrogenase, aspartate aminotransferase and alanine aminotransferase, values were significantly higher in COVID-19-positive patients compared to COVID-19-negative patients.

When we removed pre-prints, but not peer-reviewed papers, the level of heterogeneity remained above 70% across all studies (see Table S8), but the standard mean differences were statistically significant with only two exceptions (C reactive protein and procalcitonin). Biomarkers such as white blood cell, neutrophil, lymphocyte, eosinophil, platelet count, total bilirubin, albumin, and creatinine showed values significantly lower in COVID-19-positive compared to negative patients. On the other hand, biomarkers such lactate dehydrogenase, aspartate aminotransferase, alanine aminotransferase, blood urea nitrogen, D-Dimer FEU values were statistically significantly higher in COVID-19-positive compared to -negative patients.

## 4. Discussion

In this meta-analysis of routine laboratory biomarkers, we found that white blood cell, neutrophil, lymphocyte, eosinophil and platelet counts were decreased in COVID-19-positive patients, while lactate dehydrogenase, aspartate aminotransferase and alanine aminotransferase were increased in COVID-19-positive patients in a hospital setting.

Previous attempts have been made using routine laboratory biomarkers for diagnosing COVID-19. In a study of 200 hospitalised patients with suspected COVID-19, Mardani, Ahmadi and Vasmehjani [17] suggested lactate dehydrogenase, C-reactive protein, alanine aminotransferase, and neutrophil count were useful for diagnosing COVID-19. In their study, these biomarkers were significantly different in COVID-19-positive compared to -negative patients, but only lactate dehydrogenase and C-reactive protein values were outside of the normal ranges. Another study reported eosinopenia (<0.02 109/L) alone or in combination with elevated high-sensitivity C-reactive protein (≥4 mg/L) could be used for separating the two groups and thus providing a biomarker with predictive capacity for diagnosing COVID-19 [18]. A decrease in circulating eosinophils was also reported to have a good predictive value for COVID-19 and is more common in COVID-19-positive patients compared to patients with other types of pneumonia [19]. Lactate dehydrogenase and lymphocyte counts were particularly interesting, with the mean lactate dehydrogenase levels in COVID-19-positive patients raised above the normal range, while individuals with COVID-19 are often lymphopenic [20]. Lymphocytes and lactate dehydrogenase were also identified in prognostic systematic literature reviews as useful markers of severe disease [21]. However, whilst all COVID-19-positive individuals had lower mean lymphocyte counts, they were not rendered lymphopenic, meaning lymphocyte counts surprisingly had limited utility in differentiating COVID-19-positive from COVID-19-negative pneumonia patients [22]. However, the included studies were predominantly from China, where early molecular diagnostics were likely to have lacked sensitivity and had a high limit of detection. This could have introduced diagnostic bias amongst pauci-symptomatic individuals, who potentially have lower level viraemias than those with highly symptomatic infection [23]. 

Inflammatory markers may be useful in supporting the diagnosis of COVID-19 and differentiating it from other viral pneumonias. C-reactive protein is associated with overproduction of inflammatory cytokines in patients, which is linked with the degree of severity and mortality of patients with COVID-19 [24]. It was even reported as being a promising biomarker that could potentially be used for assessing disease mortality [25]. This review indicates that inflammatory markers seem unlikely to differentiate COVID-19 from bacterial pneumonia. Procalcitonin is another inflammatory marker thought to be more specific for bacterial infection. In this meta-analysis, the standard mean difference was lower in COVID-19-positive patients, but the literature is somewhat inconsistent [26,27,28,29]. Some studies reported that procalcitonin levels correlate with disease severity in COVID-19-positive patients and can, as such, help to predict the prognosis in confirmed COVID-19 cases [30]. A meta-analysis even demonstrated a ~5-fold increased risk of severe SARS-CoV-2 infection in patients with elevated procalcitonin [31]. However, concurrent bacterial infection could bias these results, and it would act as a strong confounding factor. 

The impact of these results in the emergency department will need to be further evaluated. The standard mean difference of certain biomarkers shows a statistically significant difference, but the means are often within the normative ranges. This implies that a non-negligible number of individual patients with COVID-19 would have normal levels of the biomarkers. Thus, no single biomarker will have the sensitivity and specificity to diagnose or exclude COVID-19. In parallel to our review, a Cochrane review [6] analysing the increased or decreased test results compared to normal range values was published. This review explored whether routine laboratory tests were sufficiently accurate to diagnose COVID-19 and concluded that these tests cannot accurately differentiate between COVID-19 and other diseases.

There is a suggestion that multiple biomarkers could be combined and added into a composite reference standard for diagnosing COVID-19 [32]. This option seems reasonable when considering the biomarkers identified by the current review; where low neutrophil, lymphocyte, and platelet counts are unlikely to discriminate between respiratory infections and COVID-19; but lactate dehydrogenase, aspartate aminotransferase, and alanine aminotransferase levels seem a more characteristic feature of COVID-19 [17,21,33,34,35]. These biomarkers are increased when tissues are damaged and, in particular, when the liver is affected [36]. COVID-19 does not just cause respiratory symptoms, clearly demonstrated by its ability to cause thromboembolic events, and gastrointestinal and even central nervous system infection [37,38]. However, further studies and analyses are required to compare COIVD-19 to seasonal influenza viruses, which may mimic COVID-19 infection.

Currently RT-PCR is the most commonly used reference standard [39,40], but its imperfect performance means that a composite gold standard could be used to better classify disease status [32,41]. In COVID-19, this would include not only RT-PCR but also radiology, expert opinion, and laboratory test results to correctly identify COVID-19. This method is used when only imperfect tests exist, with no established gold standard [42]; and would provide a reference to test the performance of novel COVID-19 diagnostics against. This will be of upmost importance in the immediate future, where there is an urgent desire to identify sensitive POC tests for both symptomatic and asymptomatic individuals. However, without a careful selection of an accurate gold standard, the evaluation of such diagnostics is problematic, and sensitivity can be over-estimated.

From the start of the COVID-19 outbreak in the UK, the RT-PCR testing infrastructure has expanded dramatically [43]. In the study conceptualisation phase, during the first wave there was an increase in demand for testing that lengthened the time to obtain a RT-PCR result, and the current hospital bed pressures have further placed a high demand on side rooms and isolation facilities. As the testing infrastructure continues to develop, improvements in sensitivity, the increase in high-throughput diagnostics and reductions in time to result are likely to increase the use of molecular tests at the expense of the biomarkers in the study. However, biomarkers often have the advantage of faster turn-around times, when compared to current COVID-19 diagnostics, meaning results are often available before the RT-PCR results. Clinically, this is extremely relevant, as it could be used to guide the isolation of patients with suspected COVID-19. Suspected COVID-19 patients require isolation, before being de-escalated with a negative RT-PCR result [44]. However, due to a lack of sensitivity, often patients with a high clinical suspicion of COVID-19 remain in isolation pending repeat testing, further imaging or alternative investigations. This places additional pressures on hospital infection control resources. 

The development of a clinical scoring algorithm using biomarkers to inform triage and isolation strategies upon admission could have a significant impact on infection control when resources are scarce. Biomarkers may also have a role in identifying disease severity. There is evidence that biomarkers such as D-dimer are correlated with complications such as pulmonary embolism and poor outcomes. Further research is required to see whether biomarkers be used to help predict mortality and morbidity [45].

This study has limitations, mainly related to the quality of studies carried out during a global outbreak of a previously unknown disease. The conclusions rely mainly on data collected early in the pandemic time course. Early RT-PCR tests were often in-house developed laboratory tests and/or tests lacking external validation and had poor sensitivity. Patients in the COVID-19-negative group that relied on RT-PCR negative tests might be false negatives due to the low sensitivity of the test. While the sensitivity and specificity of diagnostics has improved with more recent commercially available, validated tests, the timing of included studies mean that they may be susceptible to misclassification bias. Furthermore, the reporting of data collection protocols and details around the execution of the studies were poor, in particular, the timing of laboratory data collection. Some studies also sourced data from the same hospitals with some overlapping periods. However, the use of a control group of COVID-19-negative individuals also meeting the case definition limits potential bias. Additionally, the majority of these studies collected data when a limited number of other respiratory illnesses were circulating, therefore further studies will be required to evaluate the performance of the biomarkers in the upcoming influenza season.

We decided to include articles in pre-print to reflect the full picture of an evolving landscape during the pandemic, limiting the risk of selection bias and publication bias. However, by including pre-prints in our meta-analysis we are also aware that: (1) pre-prints might change considerably after revisions, (2) there could be mistakes in the data and in the analysis, (3) the reporting quality of these papers are generally lower compared to the peer-reviewed papers [46] and (4) some papers may never actually make it to publication in a peer-reviewed journal. By the sensitivity analysis and removing pre-prints we were able to negate all the concerns around their effect upon validity of the overall results. Finally, we included studies from very diverse geographical settings (Asia, US, Middle East, Australia, Europe). The heterogeneity of the population (i.e., definition of COVID-19-positive and -negative patients and recruiting countries) probably contributed to the high I^2^ value, which limits the conclusions we can draw from the results of this study. 

## 5. Conclusions

Decreased white blood cell, neutrophil, lymphocyte, eosinophil, and platelet counts were observed, while lactate dehydrogenase, aspartate aminotransferase, and alanine aminotransferase were elevated in COVID-19-positive patients compared to COVID-19-negative patients. Despite this, the included studies reported routine laboratory biomarker results within the normal reference ranges set by laboratories across the UK NHS, suggesting that they lack utility for diagnosing COVID-19. These biomarkers may, however, have a role when combined to feed into the development of a clinical scoring algorithm or a composite reference standard to determine the performance of novel COVID-19 diagnostics.

## Data Availability

The datasets used and/or analysed during the current study are available from the corresponding author on reasonable request.

## Supplementary Materials

The following are available online at https://www.mdpi.com/article/10.3390/v13050803/s1, Table S1: Extraction table, Table S2: Quality assessment—Risk of Bias, modified QUADAS2 tool, Table S3: PRISMA Checklist, Table S4: Basic characteristics of included studies, Table S5: Statistical analysis, Table S6: Meta-analysis of laboratory findings between COVID-19 and Non-COVID-19 patients, outliers not included, Table S7: Sensitivity analysis of laboratory findings between COVID-19 and Non-COVID-19 patients, high-risk of bias studies excluded, outliers excluded, Table S8: Sensitivity analysis of laboratory findings between COVID-19 and Non-COVID-19 patients, pre-prints excluded, outliers excluded.

## Appendix A

PICO search strategy:

- Population or Problem: Highly suspected and/or confirmed RT-PCR COVID-19-positive patients compared with controls—patients that were sick in the same period of time, but were not diagnosed with COVID-19;
- Intervention or Exposure: diagnosis;
- Context: We are including all studies that report laboratory findings (biomarkers) of hospitalised patients with COVID-19 compared to controls (healthy participants, non-infectious conditions such as bacterial infection) from the same study period;
- Study design: Retrospective studies, primary observational studies, studies where data were used to inform a mathematical model.

Research question: Are there any laboratory results (biomarkers) that can be used together with/instead of RT-PCR to inform the COVID-19 diagnosis when a patient presents at the A&E with fever?

The search criteria were limited to the biomarkers that are commonly available across NHS UK hospitals to narrow down the search:

1. exp Biomarkers/
2. (C-reactive protein or CRP) ti.ab.kw.
  exp C-Reactive Protein/
3. Leukocyte ti.ab.kw.
  exp Leukocytes/
4. Lymphocyte.ti,ab,kw.
  exp Lymphocytes/
5. (Lactate Dehydrogenase or LDH).ti,ab,kw.
  exp L-Lactate Dehydrogenase/
6. D-dimer.ti,ab,kw.
7. (ferritin or serum ferritin or FRTN).ti,ab,kw.
8. (Procalcitonin or PCT).ti,ab,kw.
  exp Procalcitonin/
9. (N-terminal proBNP or NT-proBNP).ti,ab,kw.
10. fibrin/ or fibrin fibrinogen degradation products/
11. (high-sensitivity cardiac troponin or hs-cTn).ti,ab,kw.
  Troponin T/
  Troponin I/
12. (brain natriuretic peptide or BNP).ti,ab,kw.
  Peptide Fragments/ or Natriuretic Peptide, Brain/
  N-terminal pro-B-type natriuretic peptide.ti,ab,kw.
13. exp Eosinophils/
  Eosinophil count.ti,ab,kw.
  Eosinophil.ti,ab,kw.
14. (Alanine Transaminase or ALT).ti,ab,kw.
  exp Alanine Transaminases/
15. (Aspartate Aminotransferase or AST).ti,ab,kw.
  exp Aspartate Aminotransferases/
16. exp Bilirubin/
  Bilirubin.ti,ab,kw.
17. Laboratory Parameters.ti,ab,kw.
  Laboratory Findings.ti,ab,kw.
  Laboratory Results.ti,ab,kw.

NICE guidelines for developing rapid reviews on COVID-19 were followed to construct the search strategy for OVID Medline:

1. exp coronavirus/
2. ((corona* or corono*) adj1 (virus* or viral* or virinae*)).ti,ab,kw.
3. (coronavirus* or coronovirus* or coronavirinae* or Coronavirus* or Coronovirus* or Wuhan* or Hubei* or Huanan or “2019-nCoV” or 2019nCoV or nCoV2019 or “nCoV-2019” or “COVID-19” or COVID19 or “CORVID-19” or CORVID19 or “WN-CoV” or WNCoV or “HCoV-19” or HCoV19 or CoV or “2019 novel*” or Ncov or “n-cov” or “SARS-CoV-2” or “SARSCoV-2” or “SARSCoV2” or “SARS-CoV2” or SARSCov19 or “SARS-Cov19” or “SARSCov-19” or “SARS-Cov-19” or Ncovor or Ncorona* or Ncorono* or NcovWuhan* or NcovHubei* or NcovChina* or NcovChinese*).ti,ab,kw.
4. (((respiratory* adj2 (symptom* or disease* or illness* or condition*)) or “seafood market*” or “food market*”) adj10 (Wuhan* or Hubei* or China* or Chinese* or Huanan*)).ti,ab,kw.
5. ((outbreak* or wildlife* or pandemic* or epidemic*) adj1 (China* or Chinese* or Huanan*)).ti,ab,kw.
6. “severe acute respiratory syndrome*”.ti,ab,kw.
7. or/1–6
8. limit 7 to yr = “2019-Current”

Additional records identified through other sources included general search based on reference list from references found, diagnostic studies retrieved from LOVE Platform and Living Systematic review project, as well as articles found and forwarded from other members of the team.

## Appendix B

Preferred Reporting Items for Systematic Reviews and Meta-analyses (PRISMA)
