# Supplementary material for: Utility of Routine Laboratory Biomarkers to Detect COVID-19: A Systematic Review and Meta-Analysis
Source: Viruses. 2021 Apr 30;13(5):803. doi: 10.3390/v13050803 (PMC8147047; doi:10.3390/v13050803)
Supplement: Supplementary file 1 [file viruses-13-00803-s001.zip › Table S1 Extraction table.pdf]

Table S1: Extraction table

		n	Laboratory findings	Leukocyte	Neutrophil count	Lymphocyte count	Eosinophil count	Platelet count	Prothrombin	Lactate dehydrogenase	Apartate amniotransferase	Alanine aminotransferase	Bilirubin	Albumin	Procalcitonin	C-reactive protein	Serum ferritin	Blood urea nitrogen	Serum creatinine	N- terminal pro b-type natriuretic peptide	Cardiac troponin	Blood oxygen saturation	D-dimer	Estimated glomerular [illegible]
Study	COVID 19		Mean [STD]																					
			Median [IQR]																					
			n above normal range																					
			n within the normal range																					
			n below normal range																					
			threshold																					
			n above the threshold																					
			n below the threshold																					
	Non-COVID 19		Mean [STD]																					
			Median [IQR]																					
			n above normal range																					
			n within the normal range																					

			n below normal range																					
			threshold																					
			n above the threshold																					
			n below the threshold																					

Forms originally used to extract results from the selected studies, that were then collated and summarised to be then imputed in RevMan and further analysed.
