# Supplementary material for: Utility of Routine Laboratory Biomarkers to Detect COVID-19: A Systematic Review and Meta-Analysis
Source: Viruses. 2021 Apr 30;13(5):803. doi: 10.3390/v13050803 (PMC8147047; doi:10.3390/v13050803)
Supplement: Supplementary file 1 [file viruses-13-00803-s001.zip › Table S2 Quality assessment - Risk of Bias, modified QUADAS2 tool.pdf]

Table S5: Quality assessment - Risk of Bias, modified QUADAS2 tool

	Patient selection						Testing			Flow and timing			
**Study**	**Q1**	**Q2**	**Q3**	**Q4**	**Q5**	**Q6**	**Q7**	**Q8**	**Q9**	**Q10**	**Q11**	**Total**	**Overall risk of bias**
**Brinati et al. [1]**	Yes	Yes	Yes	Yes	Yes	No	No thesholds	Unclear	Yes	Unclear	Yes	7	Low
**Chen, et al. [2]**	Yes	No	Yes	Yes	Yes	No	No	Yes	Yes	Yes	Yes	8	Low
**Chen, et al. [3]**	Unclear	No	Yes	Yes	Yes	No	No	Yes	Unclear	Yes	Yes	6	High
**Cheng, et al. [4]**	Yes	Yes	Yes	Yes	Yes	No	No	Yes	Yes	Yes	Yes	9	Low
**Chi et al. [5]**	Unclear	No	Yes	Unclear	Yes	No	No thesholds	Yes	Yes	Unclear	Yes	5	High
**de Moraes Batista et al.[6]**	Yes	Yes	Yes	Yes	Yes	No	No thesholds	Yes	Yes	Yes	Yes	9	Low
**Fan et al. [7]**	No	No	Yes	No	No	No	No thesholds	No	No	Unclear	Yes	2	High
**Fei et al.[8]**	Yes	No	Yes	No	Yes	No	No thesholds	Yes	Yes	Yes	Yes	7	Low
**Feng, et al. [9]**	Yes	Yes	Yes	Yes	No	No	Yes	Yes	Yes	Yes	Yes	9	Low
**Ferrari et al.[10]**	Yes	No	Yes	No	Yes	No	No thesholds	Yes	Yes	Yes	Yes	7	Low
**Gao et al.[11]**	Yes	Yes	Yes	Yes	Yes	Yes	No thesholds	Yes	Yes	Yes	Yes	9	Low
**Han, et al. [12]**	Yes	Yes	Yes	Yes	Yes	No	Yes	Yes	Yes	Yes	Yes	10	Low
**Hsih, et al. [13]**	Unclear	Unclear	No	No	Yes	No	Yes	Yes	Yes	Yes	Yes	6	High
**Huang, et al. [14]**	Yes	Yes	Yes	Unclear	Yes	No	No thesholds	Yes	Yes	Yes	Yes	8	Low
**Kurstjens et al. [15]**	Yes	No	No	No	No	No	Yes	Yes	Yes	Yes	Yes	6	High
**Li, et al. [16]**	No	No	Yes	Yes	Yes	No	No tresholds	Yes	Yes	Yes	Yes	7	Low

**Li, et al. [17]**	No	No	Yes	Yes	Yes	No	No	Yes	Yes	Yes	Yes	7	Low
**Liang, et al. [18]**	Yes	Yes	No	No	No	No	No tresholds	Yes	Yes	Yes	Yes	6	High
**Luo, et al. [19]**	Yes	Yes	Yes	Yes	No	No	No thesholds	No	Yes	Yes	No	6	High
**Ma et al.[20]**	Unclear	Yes	Yes	Yes	Yes	No	No	Yes	Unclear	Unclear	Yes	6	High
**Mardani, et al. [21]**	Yes	Yes	Yes	Yes	Yes	No	No thesholds	Yes	Yes	Yes	Yes	9	Low
**Martín-Sánchez et al.[22]***	Yes	Unclear	Yes	Unclear	Yes	No	No thesholds	Yes	Yes	Yes	Yes	7	Low
**Mei et al.[0.]**	No	No	Yes	Yes	Yes	No	No tresholds	Yes	Yes	Yes	Yes	7	Low
**Meng et al. [24]**	Yes	No	Yes	Yes	Yes	No	No thesholds	Yes	Yes	Unclear	Yes	7	Low
**O'Reilly, et al. [25]**	Yes	Yes	Yes	No	Yes	Unclear	No	Yes	Yes	Yes	Yes	8	Low
**Peng, et al. [26]**	Yes	Yes	Yes	Yes	Yes	No	No	Yes	Yes	Yes	Yes	9	Low
**Peng, et al. [27]**	No	No	Yes	Yes	Yes	No	No tresholds	Yes	Yes	Yes	Yes	7	Low
**Pisapia et al.[28]**	Yes	Yes	Yes	No	Yes	No	No thesholds	Yes	Yes	Yes	Yes	8	Low
**Qin et al. [29]**	Unclear	Yes	Yes	No	Yes	No	No thesholds	Unclear	Yes	Yes	Yes	6	High
**Santotoribio et al.[30]**	Yes	Yes	Yes	No	Yes	No	No thesholds	Yes	Yes	Yes	Yes	8	Low
**Song, et al. [31]**	Yes	Yes	Yes	No	Yes	No	No	Yes	Yes	Yes	Yes	8	Low
**Tordjman, et al. [32]**	Yes	No	Yes	Yes	Yes	Yes	No	Yes	Yes	Yes	Yes	8	Low
**Vieceli et al.[33]**	Yes	Yes	Yes	No	Yes	No	No thesholds	Yes	Yes	Yes	Yes	8	Low
**Wang, et al. [34]**	Unclear	Unclear	Yes	Yes	Yes	No	No tresholds	Yes	Yes	Yes	Yes	7	Low
**Wu, et al. [35]**	No?	No?	Yes	Unclear	Yes	No	No tresholds	Yes	Yes	Yes	Yes	6	High
**Zhao, et al.[36]**	Unclear	Unclear	Yes	Yes	Yes	No	No	Yes	Yes	Unclear	Yes	6	High

**Zhao, et al.[37]**	Unclear	No	Yes	No	Yes	No	No thesholds	No	Yes	Unclear	No	3	High
**Zhu, et al. [38]**	Yes	Yes	Yes	Yes	Yes	No	No	Yes	Yes	Yes	Yes	9	Low

Footnotes: Risk of bias questions: Q1. Was a consecutive or random sample of patients enrolled? **Q2.** Was a case-control design avoided? **Q3.** Were laboratory findings recorded for all patients (not only for a specific subgroup)? **Q4.** Did the study avoid inappropriate exclusions? **Q5.** Were they testing only patients 'suspected COVID-19'? **Q6.** Is the reference standard likely to correctly classify the target condition? Use NO if they did not use the composite reference standard (RT-PCR+ imaging). **Q7.** If thresholds were used, were they equivalent to normal ranges for that test? **Q8.** Was the conduct or interpretation of the tests unbiased? **Q9.** Were tests performed at the same point in time for all patients in the sample? (eg. on admission) **Q10.** Were biomarker tests performed prior to COVID-19 treatment (including drugs)? **Q11.** Are the criteria for categorisation of patients clear and defined?
