# Supplementary material for: Utility of Routine Laboratory Biomarkers to Detect COVID-19: A Systematic Review and Meta-Analysis
Source: Viruses. 2021 Apr 30;13(5):803. doi: 10.3390/v13050803 (PMC8147047; doi:10.3390/v13050803)
Supplement: Supplementary file 1 [file viruses-13-00803-s001.zip › Table S4 Basic characteristics of included studies revised.pdf]

Table S4: Basic characteristics of included studies

								COVID-19		Non-COVID-19		
**Study**	**Title**	**Publication date**	**Countr y**	**Study site**	**Study period**	**Sample size**	**Confirmed positive cases [%]**	**Age**	**Male [%]**	**Age**	**Male [%]**	**Comments**
**Brinati et al. [1]**	Detection of COVID-19 Infection from Routine Blood Exams with Machine Learning: A Feasibility Study	01/07/2020	Italy	IRCCS San Raffaele Hospital (Milan, Italy)	end of Feb 2020 to mid of Mar 2020	279	63.4%	62.6±16.3	48.4	59.2±21.7	18.9	PUBLISHED
**Chen, et al. [2]**	A diagnostic model for coronavirus disease 2019 (COVID-19) based on radiological semantic and clinical features: a multi-center study	16/04/2020	China	5 independent hospitals from 4 cities (Huizhou, Shantou, Yongzhou, Meizhou)	Jan 1 to Feb 8, 2020	136	51%	42.9±13.3	59	46.7±25	65	PUBLISHED
**Chen, et al. [3]**	Differences Between COVID-19 and Suspected Then Confirmed SARS-CoV-	01/04/2020	China	Chongqing Three Gorges Central	Jan 23 to Feb 5, 2020	104	75%	45 [15-79]	50	61[28-95]	50	PUBLISHED

	2-negative Pneumonia: A Retrospective Study From a Single Center			Hospital, Chongqing								
**Cheng, et al. [4]**	Clinical Features and Chest CT Manifestations of Coronavirus Disease 2019 (COVID-19) in a Single-Center Study in Shanghai, China	27/02/2020	China	Ruijin Hospital, Shanghai	Jan 19 to Feb 6, 2020	33	33%	50.36±15.50	72.7	43.59±16.02	31.8	PUBLISHED
**Chi et al. [5]**	Differential diagnosis for suspected cases of coronavirus disease 2019: a retrospective study	18/09/2020	China	Wenzhou Central Hospital	Jan 21 to Feb 20, 2020	68	25%	53.5±13.4	52.9	41.3±17.9	66.7	PUBLISHED
**de Moraes Batista et al.[6]**	COVID-19 diagnosis prediction in emergency care patients: a machine learning approach	14/04/2020	Brazil	Hospital Israelita Albert Einstein in São Paulo, Brazil	Mar 17 to Mar 30, 2020	235	43.0	49.4±14.5	65.7	48±16.6	39.8	PRE-PRINT
**Fan et al. [7]**	Association Between ABO Blood Group System and	21/07/2020	China	Zhongnan Hospital of	Jan 1 to Mar 5, 2020	208	51%	56.8±18.3	52.4	54.0±15.0	54.4	PUBLISHED

	COVID-19 Susceptibility in Wuhan			Wuhan University								
**Fei et al.[8]**	Clinical laboratory characteristics in patients with suspected COVID-19: one single institution experience	18/09/2020	US	University of Alabama at Birmingham	Mar 28 to Jun 27, 2020	50	48%	65.4±9.6	62.5	56.3±14.0	46.2	PUBLISHED
**Feng, et al. [9]**	A Novel Triage Tool of Artificial Intelligence Assisted Diagnosis Aid System for Suspected COVID-19 Pneumonia in Fever Clinics	20/03/2020	China	Chinese People's Liberation Army General Hospital (PLAGH) in Beijing	Jan 14 to Feb 26, 2020	164	20%	39[37-41.5]	71.4	40[32.5-54.5]	63.2	PRE-PRINT
**Ferrari et al.[10]**	Routine blood tests as a potential diagnostic tool for COVID-19	06/07/2020	Italy	San Raffaele Hospital (Milan, Italy)	Feb 20 to Mar 20, 2020	207	50.7	61.8 ± 16.4	70.5	59.2 ± 21.7	52	PUBLISHED
**Gao et al.[11]**	Improving the early diagnosis of suspected patients with COVID-19: a	30/06/2020	China		Jan 29 to Feb 21, 2020	106	50%	43.5 (33.0-55.8)	47.1	30 (21.5-40.5)	66	PUBLISHED

	retrospective study of 106 patients											
**Han, et al. [12]**	Prominent Changes in Blood Coagulation of Patients With SARS-CoV-2 Infection	16/03/2020	China	Renmin Hospital, Wuhan	Jan 31 to Feb 10, 2020	134	70%	N/A	51	N/A	70	PUBLISHED
**Hsih, et al. [13]**	Featuring COVID-19 Cases via Screening Symptomatic Patients With Epidemiologic Link During Flu Season in a Medical Center of Central Taiwan	13/03/2020	Taiwa n	China Medical University Hospital, Taiwan	Jan 20 to Feb 19, 2020	43	5%	45(39-51)	50	34(3-68)	39	PUBLISHED
**Huang, et al. [14]**	A novel risk score to predict diagnosis with Coronavirus Disease 2019 (COVID-19) in suspected patients: A retrospective, multi-center, observational study	08/06/2020	China	West China Hospital of Sichuan University	Jan 21 to Feb 7, 2020	475	71%	43[32-54]	54	34[26-49]	59	PUBLISHED
**Kurstjens et al. [15]**	Rapid identification of SARS-CoV-2-infected patients at the emergency	29/06/2020	the Nether lands	multicentre	Feb 27, 2020 onwards	967	69%	70 ± 12	64.1	62 ± 16	43	PUBLISHED

	department using routine testing											
**Li, et al. [16]**	Eosinopenia and elevated C-reactive protein facilitate triage of COVID-19 patients in fever clinic: A retrospective case-control study	25/05/2020	China	Fever Clinic at Wuhan Union Hospital, Wuhan, China	Jan 31 to Feb 21, 2020	3323	14%	59.5 [48-68]	44.3	50.0[37-62]	44.8	PUBLISHED
**Li, et al. [17]**	Comparison of the clinical characteristics between RNA positive and negative patients clinically diagnosed with 2019 novel coronavirus pneumonia	23/02/2020	China	Wuhan Fourth Hospital	Jan to Feb, 2020	54	57%	54±13	48	49±12	30	PUBLISHED
**Liang, et al. [18]**	Prevalence and clinical features of 2019 novel coronavirus disease (COVID-19) in the Fever Clinic of a teaching hospital in Beijing: a single-center, retrospective study	28/02/2020	China	Peking University Third Hospital, Beijing	Jan 21 to Feb 15, 2020	88	24%	42 [34.5-66]	52.4	N/A	N/A	PRE-PRINT

**Luo, et al. [19]**	CT differential diagnosis of COVID-19 and non-COVID-19 in symptomatic suspects: a practical scoring method	07/05/2020	China	Shenzhen Hospital	Jan 10 to Feb 28, 2020	91	33%	54 [36-64]	47	37[32–47]	54	PUBLISHED
**Ma et al.[20]**	Development and validation of a risk stratification model for screening suspected cases of COVID-19 in China	31/07/2020	China	First Affiliated Hospital, College of Medicine, Zhejiang University and Taizhou Enze Medical Center (Group), Enze Hospital	Jan 23 to Feb, 2020	523	11%	47 (38-56)	66	32 (23-42)	52	PUBLISHED
**Mardani, et al. [21]**	Laboratory Parameters in Detection of COVID-19 Patients with Positive RT-PCR; a Diagnostic Accuracy Study	04/04/2020	Iran	Behpooyan Clinic Medical Center, Tehran	Feb 22 to Mar 14, 2020	200	35%	Pooled data reported: 41.3± 14.6 [19-78] (53% male)				PUBLISHED

**Martín-Sánchez et al.[22]***	Diagnostic groups and short-term outcomes in suspected COVID-19 cases treated in an emergency department	11/06/2020	Spain	Hospital Clínico San Carlos in Madrid, Spain	Feb 28 to Mar 31, 2020	1993	59%	57.8±19.3	48	50.7±20.2	37.4	PUBLISHED
**Mei et al.[0.]**	Artificial intelligence-enabled rapid diagnosis of COVID-19 patients	19/05/2020	China	multicentre	Jan 17 to Mar 3, 2020	905	46%	43.0 ± 16.4	49.8	38.6 ± 16.3	57.6	PUBLISHED
**Meng et al. [24]**	Development and utilization of an intelligent application for aiding COVID-19 diagnosis	21/03/2020	China	West China Hospital, Sichuan	Dec 20, 2019 to Feb 10, 2020	620	49%	46±55.73	68.93	68.5±77.8 1	72.59	PRE-PRINT
**O'Reilly, et al. [25]**	Epidemiology and clinical features of emergency department patients with suspected COVID -19: Initial results from the COVID-19 Emergency Department Quality Improvement Project (COVED -1)	07/05/2020	Austra lia	The Alfred Emergency and Trauma Centre	Apr 1 to Apr 14, 2020	240	5%	51±18	73	61±21	55	PUBLISHED

**Peng, et al. [26]**	Improved Early Recognition of Coronavirus Disease-2019 (COVID-19): Single-Center Data From a Shanghai Screening Hospital	01/04/2020	China	Zhoupu Hospital, Shanghai	Jan 23 to Feb 16, 2020	86	13%	40.73±11.32	45.5	39.67±13.90	45.3	PUBLISHED
**Peng, et al. [27]**	Artificial intelligence application in COVID-19 diagnosis and prediction	27/02/2020	China	Taizhou Public Hygiene in Taizhou Hospital, of Zhejiang province	Jan 17 to Feb 1, 2020	117	27%	43[34-55]	65.6	39[23-49]	56.5	PRE-PRINT
**Pisapia et al.[28]**	Differences among confirmed and not-confirmed COVID-19 patients at "D.Cotugno" hospital, Naples (Italy): what we learned from first suspected cases?	01/06/2020	Italy	"D. Cotugno" Hospital in Naples	Feb 10 to Mar 10, 2020	37	56%	49±58	65	29±11	65	PUBLISHED
**Qin et al. [29]**	A predictive model and scoring system combining clinical and CT	01/07/2020	China	Shanghai Municipal CDC	Jan 19 to Feb 6, 2020	168	52.4	47.3 ± 12.4	47.7	42.8 ± 17.3	37.5	PUBLISHED

	characteristics for the diagnosis of COVID-19											
**Santotoribio et al.[30]**	Evaluation of Routine Blood Tests for Diagnosis of Suspected Coronavirus Disease 2019	09/09/2020	Spain	Virgen del Rocio University Hospital (Seville, Spain)	Mar 26 to Apr 8, 2020	203	46%	64.6±14.9	53.2	58.5±18.7	47.7	PUBLISHED
**Song, et al. [31]**	COVID-19 early warning score: a multi-parameter screening tool to identify highly suspected patients	08/03/2020	China	First Affiliated Hospital, Zhejiang	Jan 20 to Feb 5, 2020	304	30%	53[43.5-62]	63	34[29-49]	37	PRE-PRINT
**Tordjman, et al. [32]**	Pre-test probability for SARS-Cov-2-related Infection Score: the PARIS score	17/12/2020	France	ED at Cochin Hospital (Paris, France)	Mar 15 to 5 Apr, 2020	112	45%	60.8±18.1	30	54.1±18.6	25	PUBLISHED
**Vieceli et al.[33]**	A predictive score for COVID-19 diagnosis using clinical, laboratory and chest image data	30/07/2020	Brazil	Hospital de Clínicas de Porto Alegre	Mar 17 to Apr 10, 2020	100	29%	62 (56–69)	51.7	54 (34–68)	39.4	PUBLISHED
**Wang, et al. [34]**	Development and Validation of a Diagnostic Nomogram	06/04/2020	China	PRIMARY COHORT:	Jan 25 to Mar 3, 2020	294	53%	48.4±12.4	61	50±17.6	43	PRE-PRINT

	to Predict COVID-19 Pneumonia			Second Affiliated Hospital and Yuying Children's Hospital of Wenzhou Medical University (Wenzhou, China); VALIDATION COHORT: People's Hospital of Yueqing (Yueqing, China)								
**Wu, et al. [35]**	Rapid and accurate identification of COVID-19 infection through machine	06/04/2020	China	Lanzhou Pulmonary Hospital, the First	Not reported	253	42%	47[33-64]	51.9	PNEUMONIA: 63[47-75],	52.7	PRE-PRINT

	learning based on clinical available blood test results			Hospital of Lanzhou University, Lanzhou University Second Hospital, the First People's Hospital of Lanzhou City, and Gansu Provincial Hospital						TUBERCOLOSIS: 54[33-68], LUNG CANCER: 61[54-69]		
**Zhao, et al.[36]**	A Comparative Study on the Clinical Features of COVID-19 Pneumonia to Other Pneumonias	12/03/2020	China	Second Affiliated Hospital of Anhui Medical University and Suzhou Municipal	Jan 23 to Feb 5, 2020	34	56%	48[27-56]	57.9	35[27-46]	40	PUBLISHED

				Hospital, Anhui								
**Zhao, et al.[37]**	A comparative study of the laboratory features of COVID-19 and other viral pneumonias in the recovery stage	21/07/2020	China	Xi'an Chest Hospital (Shaanxi Province of China)	Jan 31 to Apr 3, 2020	93	50%	52(35-63)	40.4	42(31-56.5)	53.3	PUBLISHED
**Zhu, et al. [38]**	Initial clinical features of suspected coronavirus disease 2019 in two emergency departments outside of Hubei, China	13/03/2020	China	The First Affiliated Hospital of USTC, Hefei	Jan 24 to Feb 20, 2020	116	28%	46[35-52]	47	35[27-53]	46	PUBLISHED

Legend: *not in English
