# Supplementary material for: Utility of Routine Laboratory Biomarkers to Detect COVID-19: A Systematic Review and Meta-Analysis
Source: Viruses. 2021 Apr 30;13(5):803. doi: 10.3390/v13050803 (PMC8147047; doi:10.3390/v13050803)

S5: Statistical analysis

Results of random-effects meta-analysis examining the pooled prevalence of combined COVID-19 positive and COVID-19 negative patients or healthy individuals (control) are presented here as a a) forest plot, b) sensitivity analysis forest plot for studies with low risk of bias, c) sensitivity analysis forest plot for published studies and d) funnel plot depicting publication bias with the overall risk of bias.

White blood cell count, x 109/L

Study or Subgroup	COVID-19 positive Mean	SD	Total	COVID-19 negative Mean	SD	Total	Weight	Std. mean difference IV, Random, 95% CI	Risk of Bias A
✔ Brinati	7.83	4.47	175	9.79	5.25	102	3.1%	-0.41 [-0.66 , -0.16]	+
✔ Chen, Xiaofeng	5.27	2.33	70	11.48	5.36	66	3.0%	-1.51 [-1.89 , -1.13]	+
✔ Chen, Xinyi	5.82	3.46	78	9.69	3.39	26	2.9%	-1.12 [-1.59 , -0.65]	−
✔ Cheng	4.2	1.54	11	5.84	2.64	22	2.6%	-0.68 [-1.43 , 0.06]	+
✔ Chi	5.27	2.08	17	6.73	1.94	51	2.8%	-0.73 [-1.29 , -0.17]	−
✔ de Moraes Batista	5.62	2.57	102	8.14	3.48	133	3.1%	-0.81 [-1.07 , -0.54]	+
✔ Fan	6.94	3.66	105	6.27	1.75	103	3.1%	0.23 [-0.04 , 0.50]	−
✔ Fei	9.37	5.93	24	9.54	6.65	26	2.8%	-0.03 [-0.58 , 0.53]	+
✔ Feng	6.28	2.42	33	7.1	3.04	45	2.9%	-0.29 [-0.74 , 0.16]	+
✔ Ferreri	6.47	2.61	105	9.79	5.25	102	3.1%	-0.80 [-1.08 , -0.52]	+
✔ Gao	5.67	2.67	53	6.94	2.81	53	3.0%	-0.46 [-0.85 , -0.07]	+
✔ Hsih	6.3	1.42	2	8.24	2.78	41	1.8%	-0.69 [-2.12 , 0.74]	+
✔ Huang	5.45	2.05	336	7.53	2.1	139	3.1%	-1.01 [-1.21 , -0.80]	−
✔ Li, Qilin	5.33	1.93	458	6.23	2.3	531	3.1%	-0.42 [-0.55 , -0.29]	−
✔ Li, Yuanyuan	4.8	2.3	31	4.2	1.5	43	2.9%	0.32 [-0.15 , 0.78]	+
✔ Liang	5.19	1.47	21	7.21	2.94	67	2.9%	-0.75 [-1.25 , -0.25]	+
✔ Luo	5.27	1.46	30	7.82	1.79	43	2.9%	-1.52 [-2.05 , -0.99]	−
✔ Ma	4.9	0.52	103	7.3	0.61	916	3.1%	-3.99 [-4.25 , -3.72]	−
✔ Mardani	4.04	1	70	6.89	1.98	130	3.0%	-1.67 [-2.00 , -1.33]	−
✔ Martín-Sánchez	6.99	4.13	1190	7.55	3.77	803	3.1%	-0.14 [-0.23 , -0.05]	+
✔ Mei	5.4	2.2	419	8.3	3.4	488	3.1%	-1.00 [-1.13 , -0.86]	+
✔ Meng	9.82	3.86	382	7.54	1.49	383	3.1%	0.78 [0.63 , 0.93]	+
✔ O'Reilly	7	3	11	10	5	229	2.8%	-0.61 [-1.21 , 0.00]	+
✔ Peng, Ling	6.41	1.57	11	7.61	2.43	75	2.8%	-0.51 [-1.14 , 0.13]	+
✔ Peng, Minfei	5.07	1.47	32	7.03	3.24	85	3.0%	-0.68 [-1.09 , -0.26]	+
✔ Pisapia	7.04	4.91	17	8.62	4.1	20	2.7%	-0.34 [-1.00 , 0.31]	+
✔ Qin	4.6	1.66	88	6.8	3.47	80	3.0%	-0.82 [-1.13 , -0.50]	−
✔ Santotoribio	22.5	23.97	94	13.7	7	109	3.1%	0.51 [0.23 , 0.79]	+
✔ Song	3.36	4.47	91	6.86	2.15	308	3.1%	-1.23 [-1.48 , -0.98]	+
✔ Vieceli	7.2	3.59	29	11.73	5.52	71	2.9%	-0.89 [-1.34 , -0.44]	+
✔ Wang	5.09	1.16	89	7.73	3.45	89	3.0%	-1.02 [-1.33 , -0.71]	+
✔ Wu	8.35	7.3	65	6.96	2.99	114	3.1%	0.28 [-0.03 , 0.58]	−
✔ Zhao, Dahai	4.6	5.1	19	7.31	7.37	15	2.7%	-0.43 [-1.11 , 0.26]	−
✔ Zhu	5.3	2.1	32	6.3	2.3	84	3.0%	-0.44 [-0.85 , -0.03]	+
**Total (95% CI)**			**4393**			**5592**	**100.0%**	**-0.67 [-0.97 , -0.37]**	

Heterogeneity: Tau² = 0.74; Chi² = 1354.07, df = 33 (P < 0.00001); I² = 98%
Test for overall effect: Z = 4.41 (P < 0.0001)
Test for subgroup differences: Not applicable

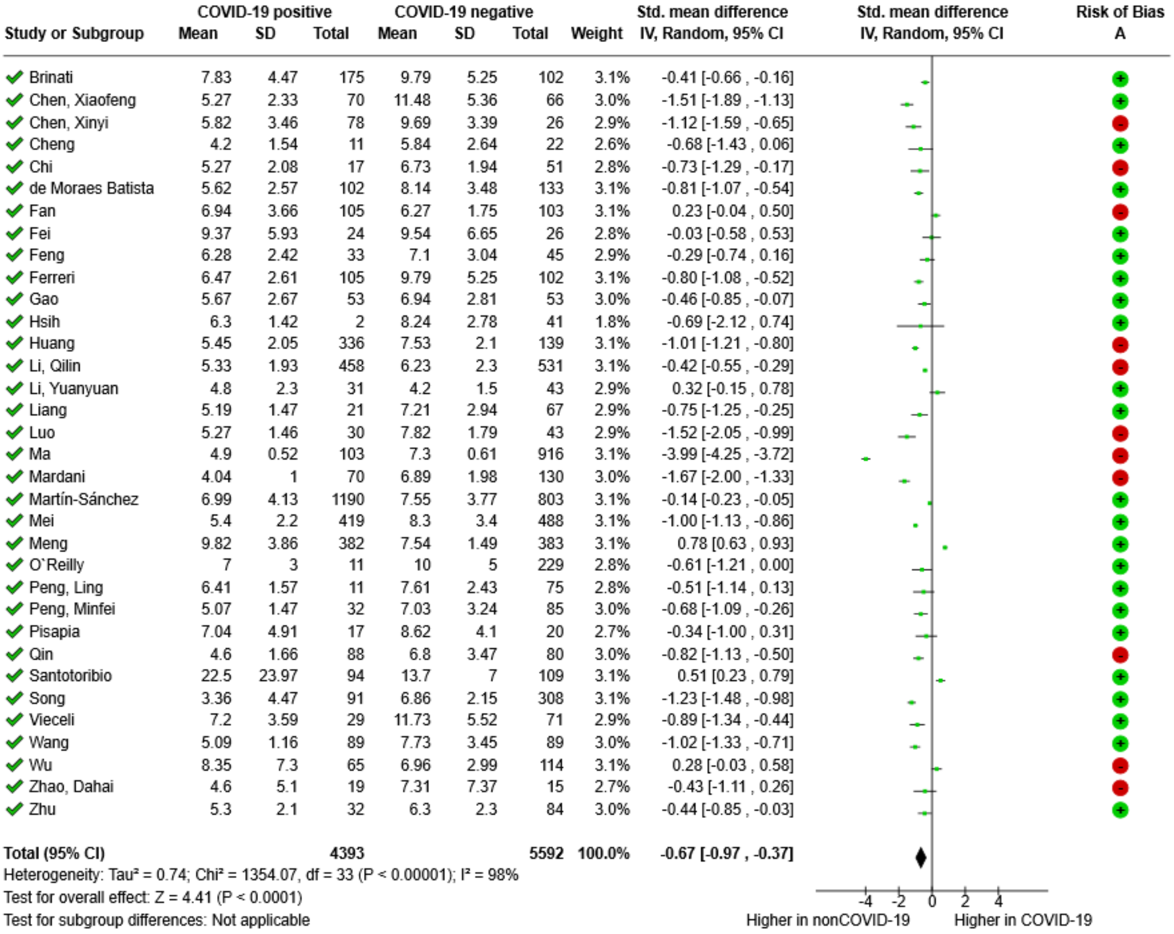

Risk of bias legend
(A) Overall risk of bias

Sensitivity analysis – low risk of bias studies

Study or Subgroup	COVID-19 positive Mean	SD	Total	COVID-19 negative Mean	SD	Total	Weight	Std. mean difference IV, Random, 95% CI	Risk of Bias A
✔ Brinati	7.83	4.47	175	9.79	5.25	102	4.7%	-0.41 [-0.66 , -0.16]	+
✔ Chen, Xiaofeng	5.27	2.33	70	11.48	5.36	66	4.5%	-1.51 [-1.89 , -1.13]	+
✖ Chen, Xinyi	5.82	3.46	78	9.69	3.39	26	0.0%	-1.12 [-1.59 , -0.65]	-
✔ Cheng	4.2	1.54	11	5.84	2.64	22	3.7%	-0.68 [-1.43 , 0.06]	+
✖ Chi	5.27	2.08	17	6.73	1.94	51	0.0%	-0.73 [-1.29 , -0.17]	-
✔ de Moraes Batista	5.62	2.57	102	8.14	3.48	133	4.7%	-0.81 [-1.07 , -0.54]	+
✖ Fan	6.94	3.66	105	6.27	1.75	103	0.0%	0.23 [-0.04 , 0.50]	-
✔ Fei	9.37	5.93	24	9.54	6.65	26	4.1%	-0.03 [-0.58 , 0.53]	+
✔ Feng	6.28	2.42	33	7.1	3.04	45	4.4%	-0.29 [-0.74 , 0.16]	+
✔ Ferreri	6.47	2.61	105	9.79	5.25	102	4.7%	-0.80 [-1.08 , -0.52]	+
✔ Gao	5.67	2.67	53	6.94	2.81	53	4.5%	-0.46 [-0.85 , -0.07]	+
✔ Hsih	6.3	1.42	2	8.24	2.78	41	2.2%	-0.69 [-2.12 , 0.74]	+
✖ Huang	5.45	2.05	336	7.53	2.1	139	0.0%	-1.01 [-1.21 , -0.80]	-
✖ Li, Qilin	5.33	1.93	458	6.23	2.3	531	0.0%	-0.42 [-0.55 , -0.29]	-
✔ Li, Yuanyuan	4.8	2.3	31	4.2	1.5	43	4.3%	0.32 [-0.15 , 0.78]	+
✔ Liang	5.19	1.47	21	7.21	2.94	67	4.3%	-0.75 [-1.25 , -0.25]	+
✖ Luo	5.27	1.46	30	7.82	1.79	43	0.0%	-1.52 [-2.05 , -0.99]	-
✖ Ma	4.9	0.52	103	7.3	0.61	916	0.0%	-3.99 [-4.25 , -3.72]	-
✖ Mardani	4.04	1	70	6.89	1.98	130	0.0%	-1.67 [-2.00 , -1.33]	-
✔ Martín-Sánchez	6.99	4.13	1190	7.55	3.77	803	4.9%	-0.14 [-0.23 , -0.05]	+
✔ Mei	5.4	2.2	419	8.3	3.4	488	4.9%	-1.00 [-1.13 , -0.86]	+
✔ Meng	9.82	3.86	382	7.54	1.49	383	4.9%	0.78 [0.63 , 0.93]	+
✔ O'Reilly	7	3	11	10	5	229	4.0%	-0.61 [-1.21 , 0.00]	+
✔ Peng, Ling	6.41	1.57	11	7.61	2.43	75	3.9%	-0.51 [-1.14 , 0.13]	+
✔ Peng, Minfei	5.07	1.47	32	7.03	3.24	85	4.4%	-0.68 [-1.09 , -0.26]	+
✔ Pisapia	7.04	4.91	17	8.62	4.1	20	3.9%	-0.34 [-1.00 , 0.31]	+
✖ Qin	4.6	1.66	88	6.8	3.47	80	0.0%	-0.82 [-1.13 , -0.50]	-
✔ Santotoribio	22.5	23.97	94	13.7	7	109	4.7%	0.51 [0.23 , 0.79]	+
✔ Song	3.36	4.47	91	6.86	2.15	308	4.7%	-1.23 [-1.48 , -0.98]	+
✔ Vieceli	7.2	3.59	29	11.73	5.52	71	4.4%	-0.89 [-1.34 , -0.44]	+
✔ Wang	5.09	1.16	89	7.73	3.45	89	4.6%	-1.02 [-1.33 , -0.71]	+
✖ Wu	8.35	7.3	65	6.96	2.99	114	0.0%	0.28 [-0.03 , 0.58]	-
✖ Zhao, Dahai	4.6	5.1	19	7.31	7.37	15	0.0%	-0.43 [-1.11 , 0.26]	-
✔ Zhu	5.3	2.1	32	6.3	2.3	84	4.5%	-0.44 [-0.85 , -0.03]	+
**Total (95% CI)**			**3024**			**3444**	**100.0%**	**-0.50 [-0.78 , -0.22]**	

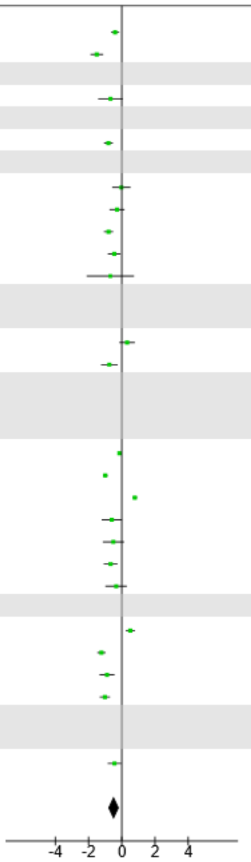

Heterogeneity: Tau² = 0.42; Chi² = 510.65, df = 22 (P < 0.00001); I² = 96%
Test for overall effect: Z = 3.48 (P = 0.0005)
Test for subgroup differences: Not applicable

Risk of bias legend
(A) Overall risk of bias

Publication bias - funnel plot

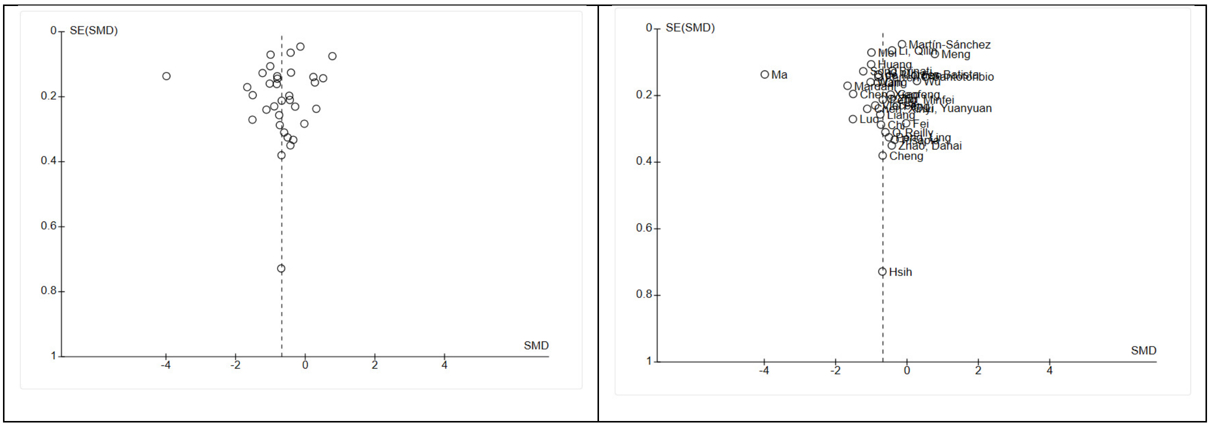

Neutrophil count, x 109/L

Study or Subgroup	COVID-19 positive Mean [x 109/L]	SD [x 109/L]	Total	COVID-19 negative Mean [x 109/L]	SD [x 109/L]	Total	Weight	Std. mean difference IV, Random, 95% CI [x 109/L]	Risk of Bias A
✔ Brinati	5.79	3.54	147	7.19	5.3	62	3.9%	-0.34 [-0.64 , -0.04]	+
✔ Chen, Xiaofeng	3.53	2.17	70	8.97	4.9	66	3.7%	-1.44 [-1.82 , -1.06]	+
✔ Chen, Xinyi	4.08	2.65	78	8.6	3.29	26	3.3%	-1.59 [-2.09 , -1.10]	−
✔ Cheng	2.94	1.4	11	3.9	2.54	22	2.5%	-0.42 [-1.15 , 0.31]	+
✔ Fei	7.54	5.37	24	7.77	6.48	26	3.1%	-0.04 [-0.59 , 0.52]	+
✔ Feng	4.29	2.43	33	5.14	2.52	19	3.1%	-0.34 [-0.91 , 0.23]	+
✔ Ferreri	4.76	2.41	94	7.18	5.3	62	3.8%	-0.63 [-0.96 , -0.30]	+
✔ Gao	3.76	2.56	53	4.78	1.99	53	3.6%	-0.44 [-0.83 , -0.06]	+
✔ Hsih	3.93	2.61	2	5.68	2.56	41	1.2%	-0.67 [-2.10 , 0.76]	+
✔ Huang	3.67	1.73	336	5.37	1.95	139	4.1%	-0.94 [-1.15 , -0.74]	−
✔ Kurstjens	5.88	3	669	8.83	5.89	298	4.3%	-0.72 [-0.86 , -0.58]	+
✔ Li, Qilin	3.67	1.71	458	4.13	1.93	531	4.3%	-0.25 [-0.38 , -0.13]	−
✔ Li, Yuanyuan	3.5	2.1	31	3	1.6	23	3.1%	0.26 [-0.28 , 0.80]	+
✔ Liang	3.39	1.48	21	5.38	2.85	67	3.3%	-0.76 [-1.26 , -0.26]	+
✔ Luo	3.59	1.03	30	5.25	2.56	43	3.3%	-0.79 [-1.28 , -0.31]	−
✔ Ma	5.58	4.13	103	5.11	0.58	916	4.1%	0.33 [0.13 , 0.54]	−
✔ Martín-Sánchez	4.7	2.46	1190	4.84	2.53	803	4.3%	-0.06 [-0.15 , 0.03]	+
✔ Mei	3.5	1.9	419	5.9	3.1	486	4.3%	-0.92 [-1.05 , -0.78]	+
✔ Peng, Minfei	3.37	1.55	32	4.87	2.87	85	3.6%	-0.58 [-0.99 , -0.16]	+
✔ Pisapia	4.75	4.72	17	6.32	3.96	20	2.8%	-0.36 [-1.01 , 0.30]	+
✔ Qin	2.93	1.43	88	4.7	3.25	80	3.9%	-0.71 [-1.03 , -0.40]	−
✔ Santotoribio	6.8	3.26	94	11.12	6.93	109	3.9%	-0.78 [-1.06 , -0.49]	+
✔ Song	5.01	4.5	91	4.53	2.2	308	4.1%	0.17 [-0.07 , 0.40]	+
✔ Tordjman	4.58	2.69	50	7.24	4.5	50	3.6%	-0.71 [-1.12 , -0.31]	+
✔ Wang	3.22	1.18	89	5.5	3.16	89	3.9%	-0.95 [-1.26 , -0.64]	+
✔ Wu	6.69	6.52	65	5.07	2.72	114	3.9%	0.36 [0.05 , 0.67]	−
✔ Zhao, Guolian	3.56	1.79	47	4.2	2.6	45	3.6%	-0.29 [-0.70 , 0.13]	−
✔ Zhu	3.7	1.9	32	4.4	2.2	84	3.6%	-0.33 [-0.74 , 0.08]	+
**Total (95% CI)**			**4374**			**4667**	**100.0%**	**-0.49 [-0.67 , -0.31]**	

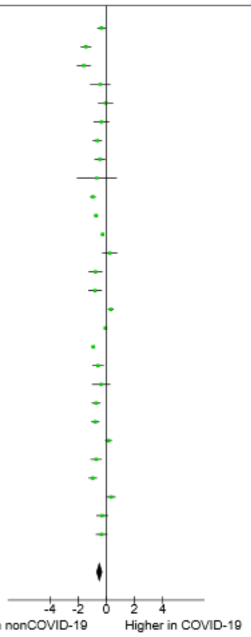

Heterogeneity: Tau² = 0.20; Chi² = 349.41, df = 27 (P < 0.00001); I² = 92%
Test for overall effect: Z = 5.32 (P < 0.00001)
Test for subgroup differences: Not applicable

Risk of bias legend
(A) Overall risk of bias

Sensitivity analysis – low risk of bias studies

Study or Subgroup	COVID-19 positive Mean [x 109/L]	SD [x 109/L]	Total	COVID-19 negative Mean [x 109/L]	SD [x 109/L]	Total	Weight	Std. mean difference IV, Random, 95% CI [x 109/L]	Risk of Bias A
✔ Brinati	5.79	3.54	147	7.19	5.3	62	5.6%	-0.34 [-0.64 , -0.04]	+
✔ Chen, Xiaofeng	3.53	2.17	70	8.97	4.9	66	5.3%	-1.44 [-1.82 , -1.06]	+
✘ Chen, Xinyi	4.08	2.65	78	8.6	3.29	26	0.0%	-1.59 [-2.09 , -1.10]	−
✔ Cheng	2.94	1.4	11	3.9	2.54	22	3.6%	-0.42 [-1.15 , 0.31]	+
✔ Fei	7.54	5.37	24	7.77	6.48	26	4.4%	-0.04 [-0.59 , 0.52]	+
✔ Feng	4.29	2.43	33	5.14	2.52	19	4.4%	-0.34 [-0.91 , 0.23]	+
✔ Ferreri	4.76	2.41	94	7.18	5.3	62	5.5%	-0.63 [-0.96 , -0.30]	+
✔ Gao	3.76	2.56	53	4.78	1.99	53	5.2%	-0.44 [-0.83 , -0.06]	+
✔ Hsih	3.93	2.61	2	5.68	2.56	41	1.6%	-0.67 [-2.10 , 0.76]	+
✘ Huang	3.67	1.73	336	5.37	1.95	139	0.0%	-0.94 [-1.15 , -0.74]	−
✔ Kurstjens	5.88	3	669	8.83	5.89	298	6.2%	-0.72 [-0.86 , -0.58]	+
✘ Li, Qilin	3.67	1.71	458	4.13	1.93	531	0.0%	-0.25 [-0.38 , -0.13]	−
✔ Li, Yuanyuan	3.5	2.1	31	3	1.6	23	4.5%	0.26 [-0.28 , 0.80]	+
✔ Liang	3.39	1.48	21	5.38	2.85	67	4.7%	-0.76 [-1.26 , -0.26]	+
✘ Luo	3.59	1.03	30	5.25	2.56	43	0.0%	-0.79 [-1.28 , -0.31]	−
✘ Ma	5.58	4.13	103	5.11	0.58	916	0.0%	0.33 [0.13 , 0.54]	−
✔ Martín-Sánchez	4.7	2.46	1190	4.84	2.53	803	6.3%	-0.06 [-0.15 , 0.03]	+
✔ Mei	3.5	1.9	419	5.9	3.1	486	6.2%	-0.92 [-1.05 , -0.78]	+
✔ Peng, Minfei	3.37	1.55	32	4.87	2.87	85	5.1%	-0.58 [-0.99 , -0.16]	+
✔ Pisapia	4.75	4.72	17	6.32	3.96	20	4.0%	-0.36 [-1.01 , 0.30]	+
✘ Qin	2.93	1.43	88	4.7	3.25	80	0.0%	-0.71 [-1.03 , -0.40]	−
✔ Santotoribio	6.8	3.26	94	11.12	6.93	109	5.7%	-0.78 [-1.06 , -0.49]	+
✔ Song	5.01	4.5	91	4.53	2.2	308	5.9%	0.17 [-0.07 , 0.40]	+
✔ Tordjman	4.58	2.69	50	7.24	4.5	50	5.2%	-0.71 [-1.12 , -0.31]	+
✔ Wang	3.22	1.18	89	5.5	3.16	89	5.6%	-0.95 [-1.26 , -0.64]	+
✘ Wu	6.69	6.52	65	5.07	2.72	114	0.0%	0.36 [0.05 , 0.67]	−
✘ Zhao, Guolian	3.56	1.79	47	4.2	2.6	45	0.0%	-0.29 [-0.70 , 0.13]	−
✔ Zhu	3.7	1.9	32	4.4	2.2	84	5.1%	-0.33 [-0.74 , 0.08]	+
**Total (95% CI)**			**3169**			**2773**	**100.0%**	**-0.51 [-0.72 , -0.30]**	

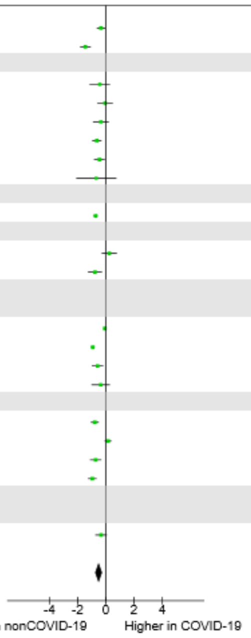

Heterogeneity: Tau² = 0.19; Chi² = 214.65, df = 19 (P < 0.00001); I² = 91%
Test for overall effect: Z = 4.69 (P < 0.00001)
Test for subgroup differences: Not applicable

Risk of bias legend
(A) Overall risk of bias

Publication bias - funnel plot

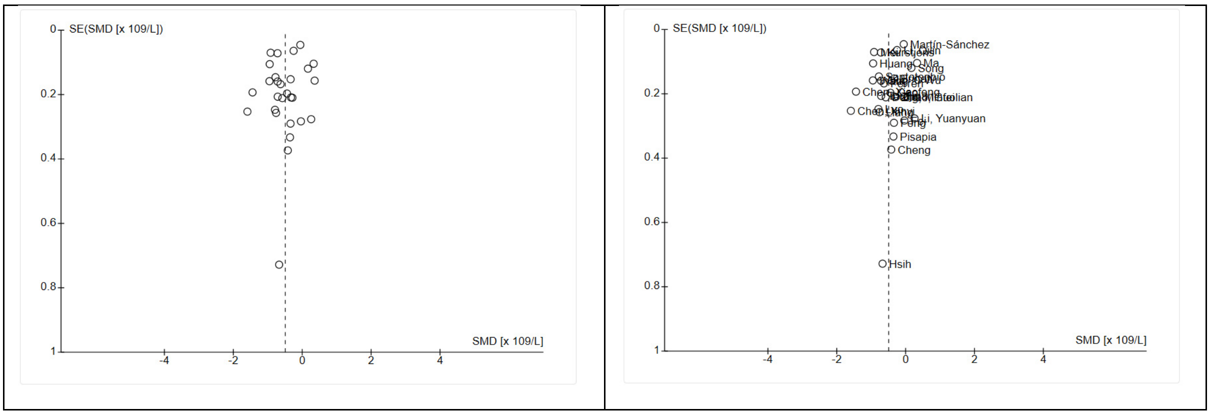

Lymphocyte count, x 109/L

Study or Subgroup	COVID-19 positive Mean [x 109/L]	SD [x 109/L]	Total	COVID-19 negative Mean [x 109/L]	SD [x 109/L]	Total	Weight	Std. mean difference IV, Random, 95% CI [x 109/L]	Risk of Bias A
Brinati	1.05	0.73	147	1.5	0.89	62	3.3%	-0.57 [-0.88 , -0.27]	+
Chen, Xiaofeng	1.25	0.68	70	1.57	1.33	66	3.2%	-0.30 [-0.64 , 0.03]	+
Chen, Xinyi	1.29	1.49	78	1.07	0.79	26	3.0%	0.16 [-0.28 , 0.61]	−
Cheng	1.15	0.25	11	1.29	0.53	22	2.2%	-0.30 [-1.03 , 0.43]	+
Chi	1.35	0.83	17	1.63	0.81	51	2.7%	-0.34 [-0.89 , 0.21]	−
de Moraes Batista	1.17	0.56	102	1.95	1.06	133	3.4%	-0.88 [-1.15 , -0.61]	+
Fan	0.81	0.47	105	1.64	0.49	103	3.3%	-1.72 [-2.04 , -1.40]	−
Fei	6.28	26.15	24	1.06	0.65	26	2.7%	0.28 [-0.27 , 0.84]	+
Feng	1.43	0.86	33	1.15	0.62	19	2.6%	0.35 [-0.22 , 0.92]	+
Ferreri	1.13	0.81	94	1.5	0.89	62	3.3%	-0.44 [-0.76 , -0.11]	+
Gao	1.36	0.62	53	1.58	0.68	53	3.1%	-0.34 [-0.72 , 0.05]	+
Hsih	1.83	1.05	2	1.5	0.77	41	1.0%	0.42 [-1.01 , 1.84]	+
Huang	1.09	0.52	336	1.37	0.52	139	3.5%	-0.54 [-0.74 , -0.34]	−
Kurstjens	1.24	3.57	669	1.5	1.03	298	3.6%	-0.09 [-0.22 , 0.05]	+
Li, Qilin	1.1	0.45	458	1.47	0.67	531	3.6%	-0.64 [-0.77 , -0.51]	−
Li, Yuanyuan	0.9	0.5	31	1	0.5	23	2.7%	-0.20 [-0.74 , 0.34]	+
Liang	1.26	0.64	21	1.19	0.62	67	2.8%	0.11 [-0.38 , 0.60]	+
Luo	1.36	0.47	30	2.04	0.84	43	2.8%	-0.94 [-1.44 , -0.45]	−
Ma	1.1	0.14	103	1.33	0.16	916	3.5%	-1.45 [-1.67 , -1.24]	−
Martín-Sánchez	0.96	0.55	1190	1.33	0.66	803	3.7%	-0.62 [-0.71 , -0.53]	+
Mei	1.4	0.8	419	1.6	0.9	486	3.6%	-0.23 [-0.36 , -0.10]	+
Peng, Ling	1.43	0.6	11	2.32	3.16	75	2.5%	-0.30 [-0.93 , 0.34]	+
Peng, Minfei	1.2	0.54	32	1.37	0.75	85	3.1%	-0.24 [-0.65 , 0.17]	+
Pisapia	1.57	0.63	17	1.41	0.62	20	2.4%	0.25 [-0.40 , 0.90]	+
Qin	1.27	0.45	88	1.4	0.68	80	3.3%	-0.23 [-0.53 , 0.08]	−
Santotoribio	14.36	20.41	94	2.01	0.89	109	3.4%	0.89 [0.60 , 1.17]	+
Song	0.82	0.51	91	1.57	0.69	308	3.4%	-1.15 [-1.39 , -0.90]	+
Tordjman	0.93	0.47	50	2.02	0.92	50	3.0%	-1.48 [-1.93 , -1.04]	+
Wang	1.32	0.38	89	1.38	0.57	89	3.3%	-0.12 [-0.42 , 0.17]	+
Wu	1.21	1.48	65	1.34	0.66	114	3.3%	-0.13 [-0.43 , 0.18]	−
Zhao, Dahai	1.1	1.39	19	1.23	1.09	15	2.3%	-0.10 [-0.78 , 0.58]	−
Zhao, Guolian	1.3	0.58	47	1.49	0.55	45	3.1%	-0.33 [-0.74 , 0.08]	−
Zhu	1.1	0.6	32	1.5	0.6	84	3.0%	-0.66 [-1.08 , -0.25]	+
**Total (95% CI)**			**4628**			**5044**	**100.0%**	**-0.40 [-0.57 , -0.23]**	

Heterogeneity: Tau² = 0.21; Chi² = 400.50, df = 32 (P < 0.00001); I² = 92%
Test for overall effect: Z = 4.55 (P < 0.00001)
Test for subgroup differences: Not applicable

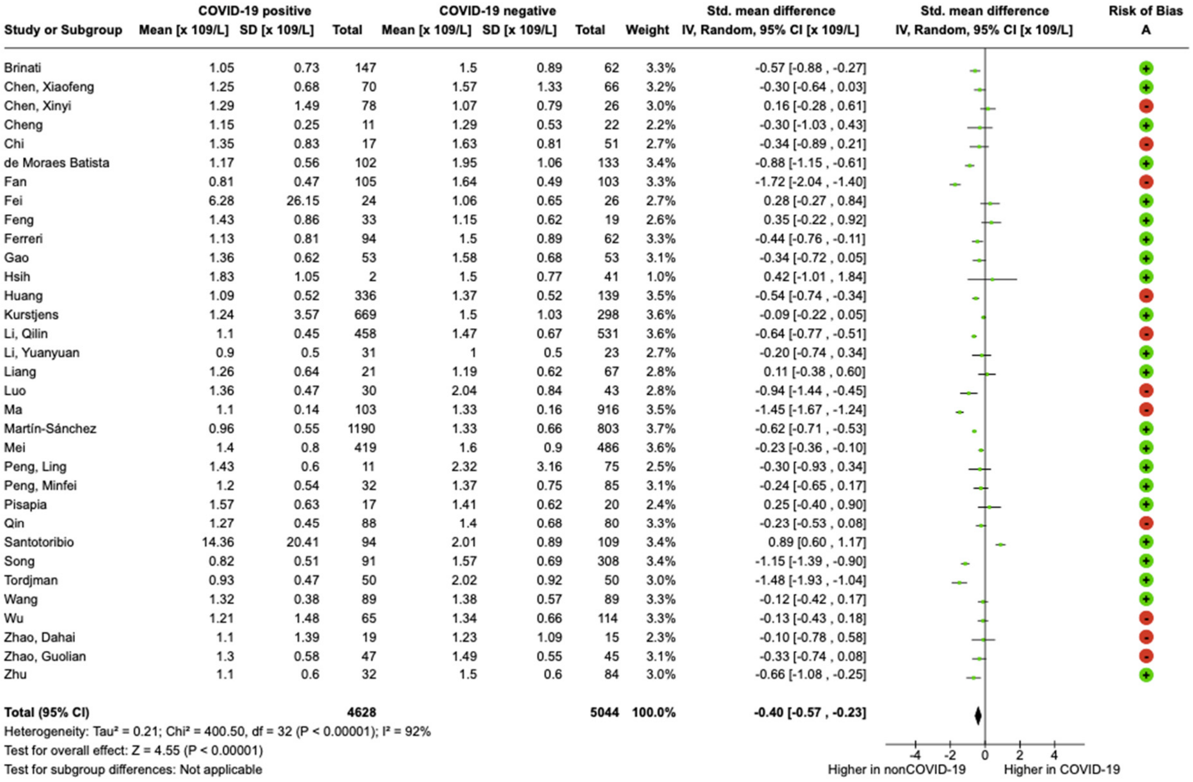

Sensitivity analysis – low risk of bias studies

Study or Subgroup	COVID-19 positive Mean [x 109/L]	SD [x 109/L]	Total	COVID-19 negative Mean [x 109/L]	SD [x 109/L]	Total	Weight	Std. mean difference IV, Random, 95% CI [x 109/L]	Risk of Bias A
✔ Brinati	1.05	0.73	147	1.5	0.89	62	5.1%	-0.57 [-0.88 , -0.27]	+
✔ Chen, Xiaofeng	1.25	0.68	70	1.57	1.33	66	5.0%	-0.30 [-0.64 , 0.03]	+
✖ Chen, Xinyi	1.29	1.49	78	1.07	0.79	26	0.0%	0.16 [-0.28 , 0.61]	−
✔ Cheng	1.15	0.25	11	1.29	0.53	22	3.3%	-0.30 [-1.03 , 0.43]	+
✖ Chi	1.35	0.83	17	1.63	0.81	51	0.0%	-0.34 [-0.89 , 0.21]	−
✔ de Moraes Batista	1.17	0.56	102	1.95	1.06	133	5.2%	-0.88 [-1.15 , -0.61]	+
✖ Fan	0.81	0.47	105	1.64	0.49	103	0.0%	-1.72 [-2.04 , -1.40]	−
✔ Fei	6.28	26.15	24	1.06	0.65	26	4.0%	0.28 [-0.27 , 0.84]	+
✔ Feng	1.43	0.86	33	1.15	0.62	19	3.9%	0.35 [-0.22 , 0.92]	+
✔ Ferreri	1.13	0.81	94	1.5	0.89	62	5.0%	-0.44 [-0.76 , -0.11]	+
✔ Gao	1.36	0.62	53	1.58	0.68	53	4.8%	-0.34 [-0.72 , 0.05]	+
✔ Hsih	1.83	1.05	2	1.5	0.77	41	1.5%	0.42 [-1.01 , 1.84]	+
✖ Huang	1.09	0.52	336	1.37	0.52	139	0.0%	-0.54 [-0.74 , -0.34]	−
✔ Kurstjens	1.24	3.57	669	1.5	1.03	298	5.7%	-0.09 [-0.22 , 0.05]	+
✖ Li, Qilin	1.1	0.45	458	1.47	0.67	531	0.0%	-0.64 [-0.77 , -0.51]	−
✔ Li, Yuanyuan	0.9	0.5	31	1	0.5	23	4.1%	-0.20 [-0.74 , 0.34]	+
✔ Liang	1.26	0.64	21	1.19	0.62	67	4.3%	0.11 [-0.38 , 0.60]	+
✖ Luo	1.36	0.47	30	2.04	0.84	43	0.0%	-0.94 [-1.44 , -0.45]	−
✖ Ma	1.1	0.14	103	1.33	0.16	916	0.0%	-1.45 [-1.67 , -1.24]	−
✔ Martín-Sánchez	0.96	0.55	1190	1.33	0.66	803	5.7%	-0.62 [-0.71 , -0.53]	+
✔ Mei	1.4	0.8	419	1.6	0.9	486	5.7%	-0.23 [-0.36 , -0.10]	+
✔ Peng, Ling	1.43	0.6	11	2.32	3.16	75	3.7%	-0.30 [-0.93 , 0.34]	+
✔ Peng, Minfei	1.2	0.54	32	1.37	0.75	85	4.7%	-0.24 [-0.65 , 0.17]	+
✔ Pisapia	1.57	0.63	17	1.41	0.62	20	3.6%	0.25 [-0.40 , 0.90]	+
✖ Qin	1.27	0.45	88	1.4	0.68	80	0.0%	-0.23 [-0.53 , 0.08]	−
✔ Santotoribio	14.36	20.41	94	2.01	0.89	109	5.2%	0.89 [0.60 , 1.17]	+
✔ Song	0.82	0.51	91	1.57	0.69	308	5.3%	-1.15 [-1.39 , -0.90]	+
✔ Tordjman	0.93	0.47	50	2.02	0.92	50	4.5%	-1.48 [-1.93 , -1.04]	+
✔ Wang	1.32	0.38	89	1.38	0.57	89	5.2%	-0.12 [-0.42 , 0.17]	+
✖ Wu	1.21	1.48	65	1.34	0.66	114	0.0%	-0.13 [-0.43 , 0.18]	−
✖ Zhao, Dahai	1.1	1.39	19	1.23	1.09	15	0.0%	-0.10 [-0.78 , 0.58]	−
✖ Zhao, Guolian	1.3	0.58	47	1.49	0.55	45	0.0%	-0.33 [-0.74 , 0.08]	−
✔ Zhu	1.1	0.6	32	1.5	0.6	84	4.6%	-0.66 [-1.08 , -0.25]	+
**Total (95% CI)**			**3282**			**2981**	**100.0%**	**-0.30 [-0.50 , -0.10]**	

Heterogeneity: Tau² = 0.18; Chi² = 224.19, df = 21 (P < 0.00001); I² = 91%
Test for overall effect: Z = 2.92 (P = 0.003)
Test for subgroup differences: Not applicable

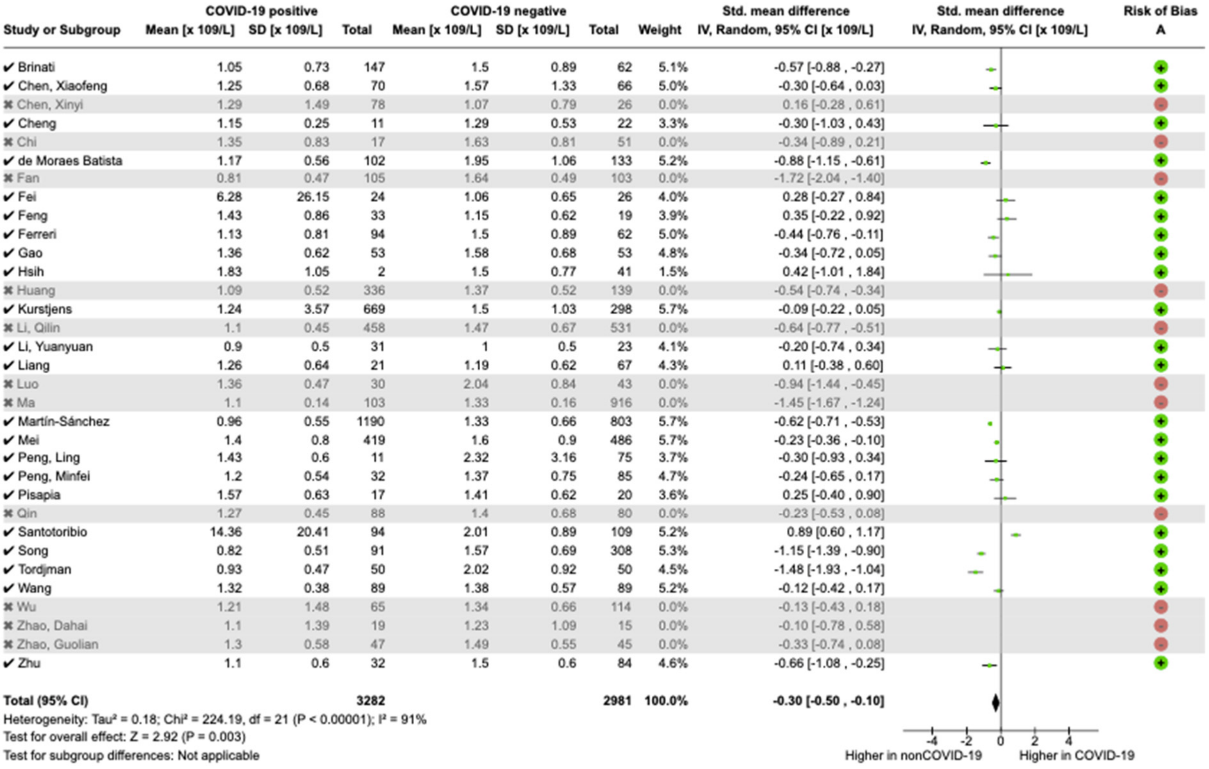

Publication bias - funnel plot

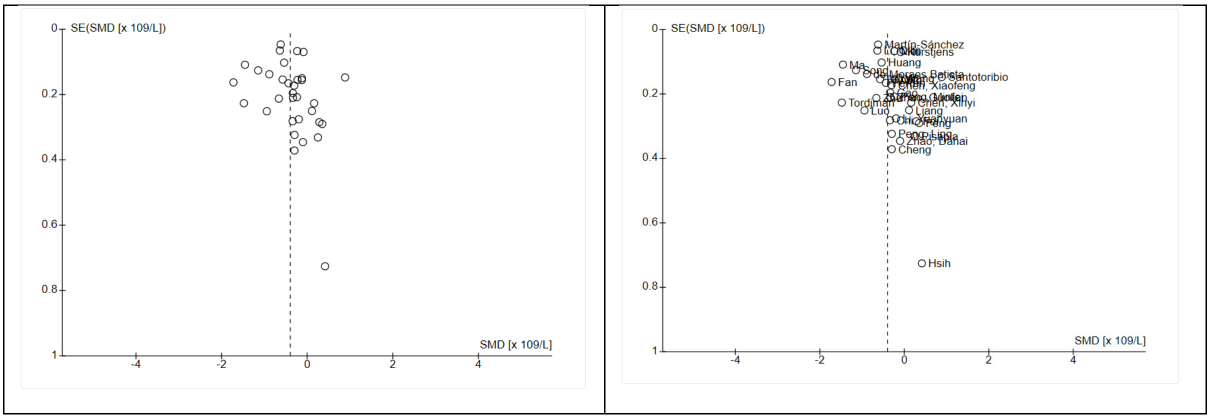

Eosinophil count, x 109/L

Study or Subgroup	COVID-19 positive Mean [x 109/L]	SD [x 109/L]	Total	COVID-19 negative Mean [x 109/L]	SD [x 109/L]	Total	Weight	Std. mean difference IV, Random, 95% CI [x 109/L]	Risk of Bias A
✔ Brinati	0.04	0.08	147	0.1	0.2	62	7.8%	-0.47 [-0.77 , -0.17]	+
✔ de Moraes Batista	0.03	0.05	102	0.18	0.21	133	7.8%	-0.93 [-1.20 , -0.65]	+
✔ Feng	0.04	0.06	33	0.07	0.07	19	7.1%	-0.46 [-1.04 , 0.11]	+
✔ Ferreri	0.02	0.06	94	0.09	0.2	62	7.7%	-0.52 [-0.84 , -0.19]	+
✔ Gao	0.04	0.06	53	0.07	0.09	53	7.6%	-0.39 [-0.77 , -0.00]	+
✔ Huang	0.01	0.02	336	0.06	0.07	139	7.9%	-1.21 [-1.42 , -0.99]	−
✔ Li, Qilin	0.01	0.01	458	0.05	0.06	531	8.0%	-0.90 [-1.03 , -0.77]	−
✔ Ma	0.01	0.01	103	0.04	0.01	916	7.9%	-3.00 [-3.24 , -2.76]	−
✔ Peng, Minfei	0.06	0.14	32	0.05	0.07	85	7.6%	0.11 [-0.30 , 0.51]	+
✔ Santotoribio	0.25	0.22	94	0.23	0.16	109	7.8%	0.10 [-0.17 , 0.38]	+
✔ Tordjman	0.03	0.1	50	0.19	0.26	50	7.5%	-0.81 [-1.21 , -0.40]	+
✔ Wu	0.04	0.04	65	0.1	0.12	114	7.8%	-0.60 [-0.92 , -0.29]	−
✔ Zhao, Guolian	0.03	0.05	47	0.09	0.08	45	7.5%	-0.90 [-1.33 , -0.47]	−
**Total (95% CI)**			**1614**			**2318**	**100.0%**	**-0.77 [-1.22 , -0.33]**	

Heterogeneity: Tau² = 0.65; Chi² = 396.30, df = 12 (P < 0.00001); I² = 97%
Test for overall effect: Z = 3.39 (P = 0.0007)
Test for subgroup differences: Not applicable

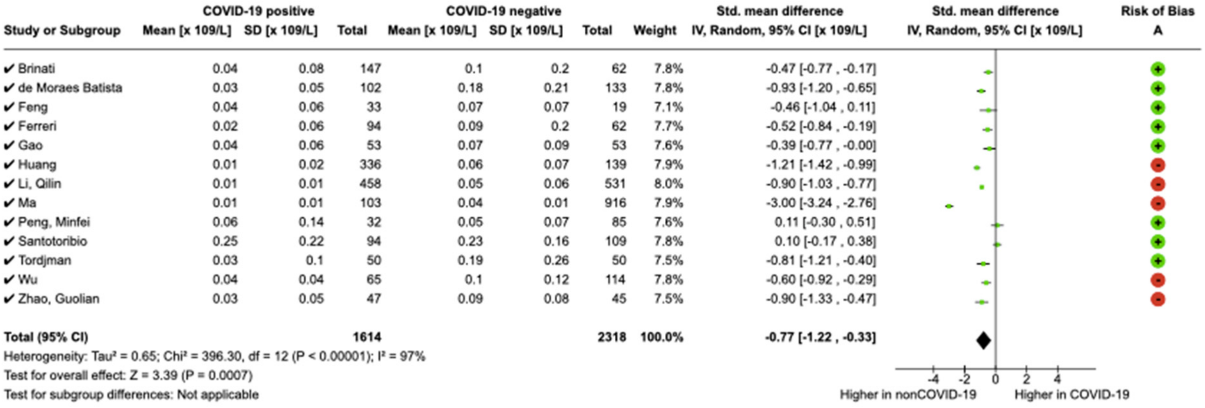

Sensitivity analysis – low risk of bias studies

Study or Subgroup	COVID-19 positive Mean [x 109/L]	COVID-19 positive SD [x 109/L]	COVID-19 positive Total	COVID-19 negative Mean [x 109/L]	COVID-19 negative SD [x 109/L]	COVID-19 negative Total	Weight	Std. mean difference IV, Random, 95% CI [x 109/L]	Risk of Bias A
✔ Brinati	0.04	0.08	147	0.1	0.2	62	13.4%	-0.47 [-0.77 , -0.17]	+
✔ de Moraes Batista	0.03	0.05	102	0.18	0.21	133	13.8%	-0.93 [-1.20 , -0.65]	+
✔ Feng	0.04	0.06	33	0.07	0.07	19	9.7%	-0.46 [-1.04 , 0.11]	+
✔ Ferreri	0.02	0.06	94	0.09	0.2	62	13.1%	-0.52 [-0.84 , -0.19]	+
✔ Gao	0.04	0.06	53	0.07	0.09	53	12.3%	-0.39 [-0.77 , -0.00]	+
✖ Huang	0.01	0.02	336	0.06	0.07	139	0.0%	-1.21 [-1.42 , -0.99]	−
✖ Li, Qilin	0.01	0.01	458	0.05	0.06	531	0.0%	-0.90 [-1.03 , -0.77]	−
✖ Ma	0.01	0.01	103	0.04	0.01	916	0.0%	-3.00 [-3.24 , -2.76]	−
✔ Peng, Minfei	0.06	0.14	32	0.05	0.07	85	12.0%	0.11 [-0.30 , 0.51]	+
✔ Santotoribio	0.25	0.22	94	0.23	0.16	109	13.8%	0.10 [-0.17 , 0.38]	+
✔ Tordjman	0.03	0.1	50	0.19	0.26	50	11.9%	-0.81 [-1.21 , -0.40]	+
✖ Wu	0.04	0.04	65	0.1	0.12	114	0.0%	-0.60 [-0.92 , -0.29]	−
✖ Zhao, Guolian	0.03	0.05	47	0.09	0.08	45	0.0%	-0.90 [-1.33 , -0.47]	−
**Total (95% CI)**			**605**			**573**	**100.0%**	**-0.42 [-0.71 , -0.13]**	

Heterogeneity: Tau² = 0.13; Chi² = 37.57, df = 7 (P < 0.00001); I² = 81%
Test for overall effect: Z = 2.88 (P = 0.004)
Test for subgroup differences: Not applicable

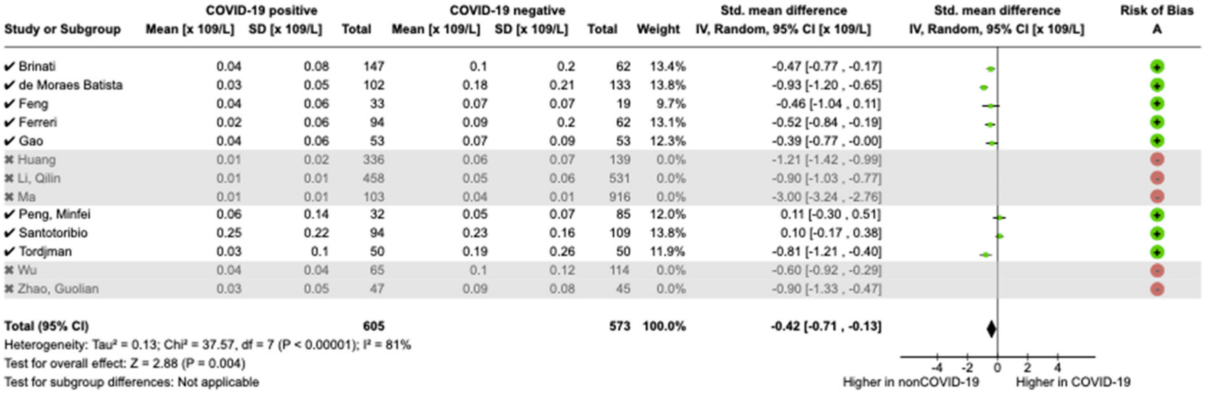

Publication bias - funnel plot

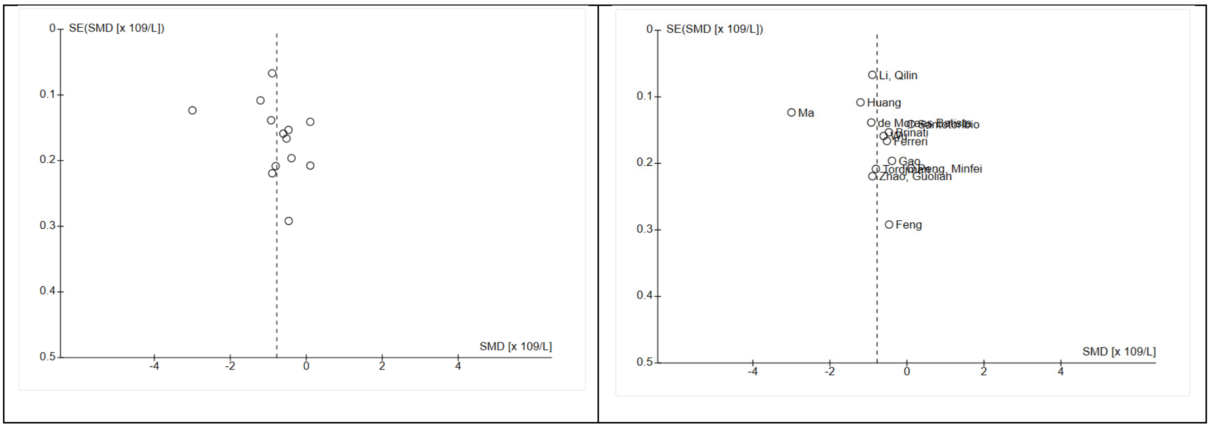

Platelet count, x 109/L

Study or Subgroup	COVID-19 positive Mean [x 109/L]	COVID-19 positive SD [x 109/L]	COVID-19 positive Total	COVID-19 negative Mean [x 109/L]	COVID-19 negative SD [x 109/L]	COVID-19 negative Total	Weight	Std. mean difference IV, Random, 95% CI [x 109/L]	Risk of Bias A
✔ Brinati	222.88	108.2	175	232.78	87.97	102	4.6%	-0.10 [-0.34 , 0.15]	+
✔ Cheng	143.5	32.63	11	209.2	57.42	22	2.7%	-1.26 [-2.06 , -0.47]	+
✔ Chi	198.41	92.02	17	217.52	72.42	51	3.5%	-0.24 [-0.79 , 0.31]	−
✔ de Moraes Batista	182.8	55.7	102	233.8	100.2	133	4.5%	-0.61 [-0.87 , -0.34]	+
✔ Fei	204.23	109.77	24	237.2	141.77	26	3.5%	-0.25 [-0.81 , 0.30]	+
✔ Feng	197.14	57.86	33	224.67	77.69	19	3.4%	-0.41 [-0.98 , 0.16]	+
✔ Ferreri	208.1	92.1	105	232.8	88	102	4.5%	-0.27 [-0.55 , 0.00]	+
✔ Gao	207.74	63.4	53	203	76.2	53	4.1%	0.07 [-0.31 , 0.45]	+
✔ Huang	172.67	56.58	336	194.33	63.68	139	4.7%	-0.37 [-0.57 , -0.17]	−
✔ Li, Qilin	180.67	56.52	458	203.67	75.82	531	4.8%	-0.34 [-0.47 , -0.21]	−
✔ Li, Yuanyuan	174	63	31	160	44	23	3.5%	0.25 [-0.29 , 0.79]	+
✔ Liang	162.6	59	21	189.8	67.8	67	3.7%	-0.41 [-0.90 , 0.08]	+
✔ Ma	186.06	20.13	103	218.41	13.48	916	4.6%	-2.26 [-2.49 , -2.04]	−
✔ Martín-Sánchez	205.49	85.92	1190	230.06	82.22	803	4.9%	-0.29 [-0.38 , -0.20]	+
✔ Meng	162.61	49.03	382	187.41	38.51	383	4.8%	-0.56 [-0.71 , -0.42]	+
✔ Peng, Minfei	185.67	74.5	32	222	50.53	85	4.0%	-0.62 [-1.04 , -0.21]	+
✔ Qin	181.33	62.18	88	222.27	75.72	80	4.4%	-0.59 [-0.90 , -0.28]	−
✔ Santotoribio	248.46	83.4	94	300.94	101.6	109	4.5%	-0.56 [-0.84 , -0.28]	+
✔ Song	189.51	64.64	91	224.17	62.29	308	4.6%	-0.55 [-0.79 , -0.31]	+
✔ Tordjman	207	91	50	270	102	50	4.0%	-0.65 [-1.05 , -0.24]	+
✔ Vieceli	205.67	56.92	29	220	81.73	71	3.9%	-0.19 [-0.62 , 0.24]	+
✔ Wang	217.67	78.37	89	235.33	72.34	89	4.4%	-0.23 [-0.53 , 0.06]	+
✔ Wu	187.88	87.07	65	206.43	85.03	114	4.4%	-0.22 [-0.52 , 0.09]	−
✔ Zhu	157.2	54.5	32	199.6	64.1	84	4.0%	-0.68 [-1.10 , -0.27]	+
**Total (95% CI)**			**3611**			**4360**	**100.0%**	**-0.47 [-0.66 , -0.28]**	

Heterogeneity: Tau² = 0.19; Chi² = 304.59, df = 23 (P < 0.00001); I² = 92%
Test for overall effect: Z = 4.85 (P < 0.00001)
Test for subgroup differences: Not applicable

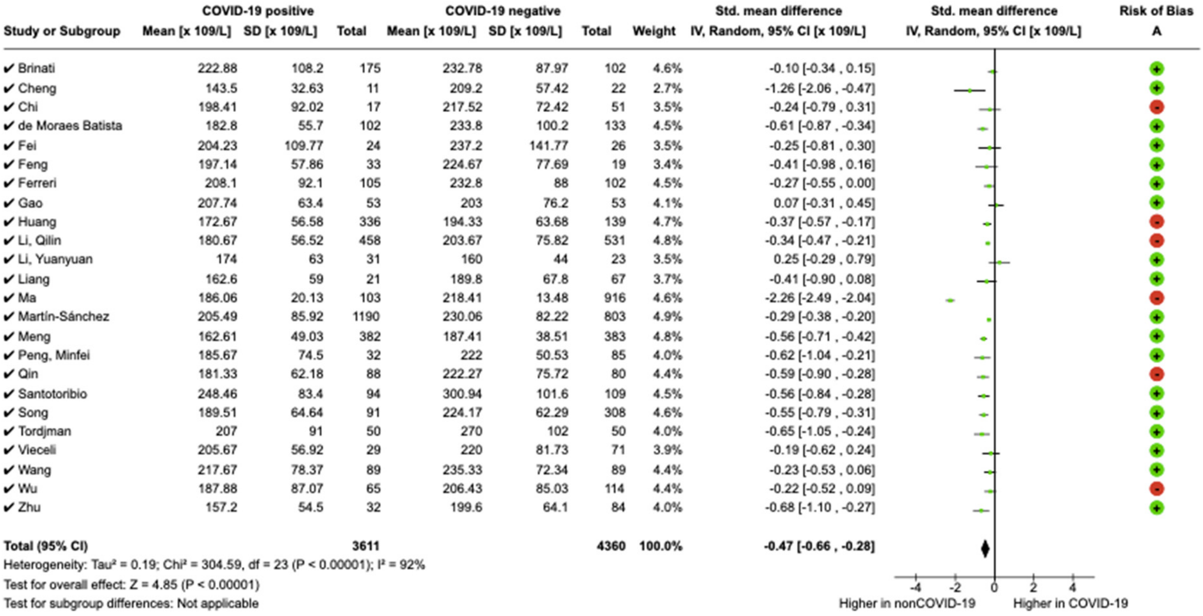

Sensitivity analysis – low risk of bias studies

Study or Subgroup	COVID-19 positive Mean [x 109/L]	SD [x 109/L]	Total	COVID-19 negative Mean [x 109/L]	SD [x 109/L]	Total	Weight	Std. mean difference IV, Random, 95% CI [x 109/L]	Risk of Bias A
✔ Brinati	222.88	108.2	175	232.78	87.97	102	7.5%	-0.10 [-0.34 , 0.15]	+
✔ Cheng	143.5	32.63	11	209.2	57.42	22	1.7%	-1.26 [-2.06 , -0.47]	+
✖ Chi	198.41	92.02	17	217.52	72.42	51	0.0%	-0.24 [-0.79 , 0.31]	−
✔ de Moraes Batista	182.8	55.7	102	233.8	100.2	133	7.1%	-0.61 [-0.87 , -0.34]	+
✔ Fei	204.23	109.77	24	237.2	141.77	26	3.0%	-0.25 [-0.81 , 0.30]	+
✔ Feng	197.14	57.86	33	224.67	77.69	19	2.9%	-0.41 [-0.98 , 0.16]	+
✔ Ferreri	208.1	92.1	105	232.8	88	102	6.9%	-0.27 [-0.55 , 0.00]	+
✔ Gao	207.74	63.4	53	203	76.2	53	5.0%	0.07 [-0.31 , 0.45]	+
✖ Huang	172.67	56.58	336	194.33	63.68	139	0.0%	-0.37 [-0.57 , -0.17]	−
✖ Li, Qilin	180.67	56.52	458	203.67	75.82	531	0.0%	-0.34 [-0.47 , -0.21]	−
✔ Li, Yuanyuan	174	63	31	160	44	23	3.2%	0.25 [-0.29 , 0.79]	+
✔ Liang	162.6	59	21	189.8	67.8	67	3.6%	-0.41 [-0.90 , 0.08]	+
✖ Ma	186.06	20.13	103	218.41	13.48	916	0.0%	-2.26 [-2.49 , -2.04]	−
✔ Martín-Sánchez	205.49	85.92	1190	230.06	82.22	803	10.7%	-0.29 [-0.38 , -0.20]	+
✔ Meng	162.61	49.03	382	187.41	38.51	383	9.6%	-0.56 [-0.71 , -0.42]	+
✔ Peng, Minfei	185.67	74.5	32	222	50.53	85	4.5%	-0.62 [-1.04 , -0.21]	+
✖ Qin	181.33	62.18	88	222.27	75.72	80	0.0%	-0.59 [-0.90 , -0.28]	−
✔ Santotoribio	248.46	83.4	94	300.94	101.6	109	6.7%	-0.56 [-0.84 , -0.28]	+
✔ Song	189.51	64.64	91	224.17	62.29	308	7.6%	-0.55 [-0.79 , -0.31]	+
✔ Tordjman	207	91	50	270	102	50	4.7%	-0.65 [-1.05 , -0.24]	+
✔ Vieceli	205.67	56.92	29	220	81.73	71	4.3%	-0.19 [-0.62 , 0.24]	+
✔ Wang	217.67	78.37	89	235.33	72.34	89	6.5%	-0.23 [-0.53 , 0.06]	+
✖ Wu	187.88	87.07	65	206.43	85.03	114	0.0%	-0.22 [-0.52 , 0.09]	−
✔ Zhu	157.2	54.5	32	199.6	64.1	84	4.5%	-0.68 [-1.10 , -0.27]	+
**Total (95% CI)**			**2544**			**2529**	**100.0%**	**-0.39 [-0.50 , -0.28]**	

Heterogeneity: Tau² = 0.03; Chi² = 44.08, df = 17 (P = 0.0003); I² = 61%
Test for overall effect: Z = 6.76 (P < 0.00001)
Test for subgroup differences: Not applicable

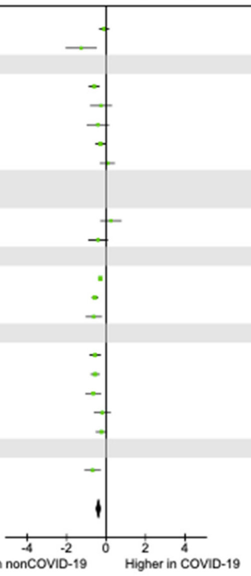

Publication bias - funnel plot

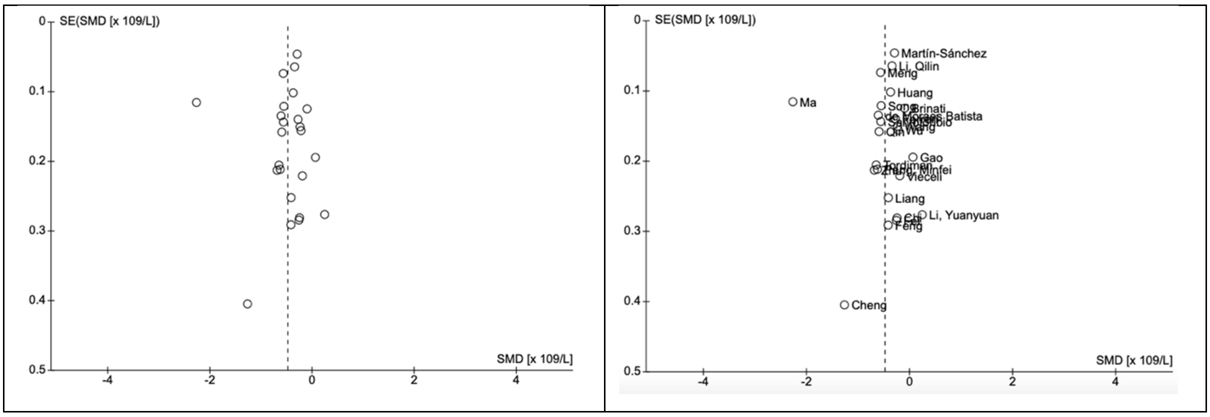

Lactate dehydrogenase (LDH), U/L

Study or Subgroup	COVID-19 positive Mean [U/L]	SD [U/L]	Total	COVID-19 negative Mean [U/L]	SD [U/L]	Total	Weight	Std. mean difference IV, Random, 95% CI [U/L]	Risk of Bias A
✔ Brinati	430.5	203.5	131	276.38	118.35	63	6.8%	0.85 [0.54 , 1.16]	+
✔ Chen, Xinyi	256.74	189.84	78	248.3	104.92	26	6.4%	0.05 [-0.40 , 0.49]	−
✔ Fei	744.17	615.75	24	407.4	253.56	26	5.9%	0.71 [0.14 , 1.29]	+
✔ Ferreri	388	154.5	78	276.4	118.3	63	6.7%	0.80 [0.45 , 1.14]	+
✔ Gao	64.98	19.37	53	241.53	57.46	53	5.5%	-4.09 [-4.77 , -3.41]	+
✔ Hsih	190	21.26	2	172.77	34.8	41	3.0%	0.49 [-0.93 , 1.91]	+
✔ Kurstjens	396.87	201.7	669	269.7	208.04	298	7.1%	0.62 [0.48 , 0.76]	+
✔ Li, Yuanyuan	250	114	31	227	88	23	6.0%	0.22 [-0.32 , 0.76]	+
✔ Mardani	465.2	100.2	70	327.6	93.2	130	6.7%	1.43 [1.11 , 1.76]	−
✔ Martín-Sánchez	631.67	97.53	1190	506.03	125.13	803	7.2%	1.15 [1.05 , 1.24]	+
✔ Santotoribio	391.79	144.33	94	300.71	102.35	109	6.8%	0.73 [0.45 , 1.02]	+
✔ Song	254.83	97.94	73	177.83	42.89	231	6.8%	1.26 [0.98 , 1.55]	+
✔ Vieceli	341	127.1	29	230.67	87.02	71	6.3%	1.09 [0.63 , 1.55]	+
✔ Wu	340.6	387.8	65	206.46	71.02	114	6.8%	0.56 [0.25 , 0.87]	−
✔ Zhao, Dahai	385.64	480.58	19	163.33	101.42	15	5.4%	0.59 [-0.10 , 1.29]	−
✔ Zhu	246.5	82.1	32	193.5	72.2	84	6.5%	0.70 [0.28 , 1.12]	+
**Total (95% CI)**			**2638**			**2150**	**100.0%**	**0.50 [0.18 , 0.83]**	

Heterogeneity: Tau² = 0.38; Chi² = 294.68, df = 15 (P < 0.00001); I² = 95%
Test for overall effect: Z = 3.02 (P = 0.003)
Test for subgroup differences: Not applicable

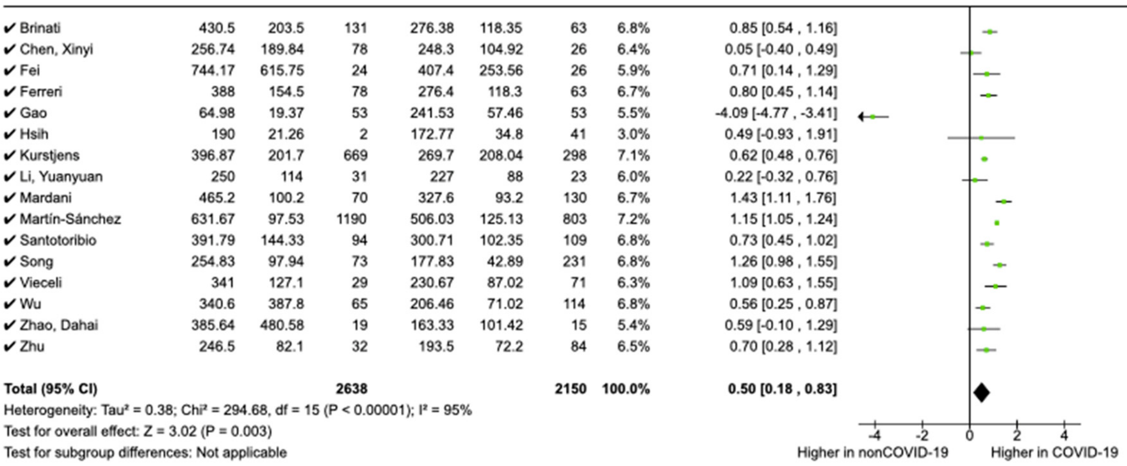

Sensitivity analysis – low risk of bias studies

Study or Subgroup	COVID-19 positive Mean [U/L]	SD [U/L]	Total	COVID-19 negative Mean [U/L]	SD [U/L]	Total	Weight	Std. mean difference IV, Random, 95% CI [U/L]	Risk of Bias A
✔ Brinati	430.5	203.5	131	276.38	118.35	63	9.0%	0.85 [0.54 , 1.16]	+
✖ Chen, Xinyi	256.74	189.84	78	248.3	104.92	26	0.0%	0.05 [-0.40 , 0.49]	−
✔ Fei	744.17	615.75	24	407.4	253.56	26	8.0%	0.71 [0.14 , 1.29]	+
✔ Ferreri	388	154.5	78	276.4	118.3	63	8.9%	0.80 [0.45 , 1.14]	+
✔ Gao	64.98	19.37	53	241.53	57.46	53	7.5%	-4.09 [-4.77 , -3.41]	+
✔ Hsih	190	21.26	2	172.77	34.8	41	4.3%	0.49 [-0.93 , 1.91]	+
✔ Kurstjens	396.87	201.7	669	269.7	208.04	298	9.4%	0.62 [0.48 , 0.76]	+
✔ Li, Yuanyuan	250	114	31	227	88	23	8.1%	0.22 [-0.32 , 0.76]	+
✖ Mardani	465.2	100.2	70	327.6	93.2	130	0.0%	1.43 [1.11 , 1.76]	−
✔ Martín-Sánchez	631.67	97.53	1190	506.03	125.13	803	9.5%	1.15 [1.05 , 1.24]	+
✔ Santotoribio	391.79	144.33	94	300.71	102.35	109	9.1%	0.73 [0.45 , 1.02]	+
✔ Song	254.83	97.94	73	177.83	42.89	231	9.1%	1.26 [0.98 , 1.55]	+
✔ Vieceli	341	127.1	29	230.67	87.02	71	8.5%	1.09 [0.63 , 1.55]	+
✖ Wu	340.6	387.8	65	206.46	71.02	114	0.0%	0.56 [0.25 , 0.87]	−
✖ Zhao, Dahai	385.64	480.58	19	163.33	101.42	15	0.0%	0.59 [-0.10 , 1.29]	−
✔ Zhu	246.5	82.1	32	193.5	72.2	84	8.6%	0.70 [0.28 , 1.12]	+
**Total (95% CI)**			**2406**			**1865**	**100.0%**	**0.44 [0.05 , 0.84]**	

Heterogeneity: Tau² = 0.42; Chi² = 264.90, df = 11 (P < 0.00001); I² = 96%
Test for overall effect: Z = 2.19 (P = 0.03)
Test for subgroup differences: Not applicable

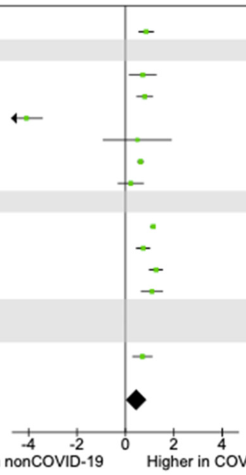

Publication bias - funnel plot

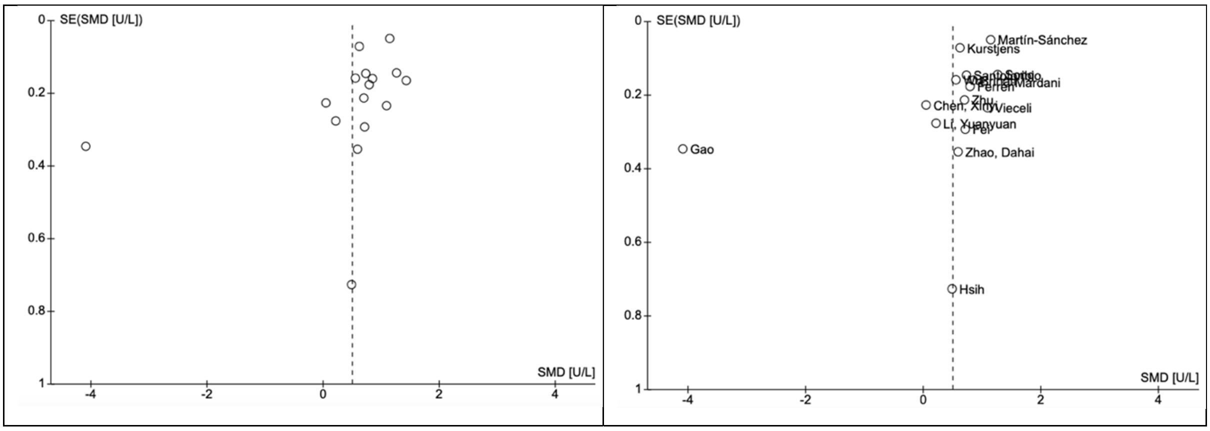

AST - Aspartate aminotransferase, U/L

Study or Subgroup	COVID-19 positive Mean [U/L]	SD [U/L]	Total	COVID-19 negative Mean [U/L]	SD [U/L]	Total	Weight	Std. mean difference IV, Random, 95% CI [U/L]	Risk of Bias A
✔ Brinati	63.61	58.13	175	38.06	53.21	102	8.4%	0.45 [0.20 , 0.70]	+
✔ Chen, Xinyi	28.97	33.87	78	31.78	25.72	26	6.0%	-0.09 [-0.53 , 0.36]	−
✔ Fei	92.63	94.7	24	61.35	80.73	26	4.8%	0.35 [-0.21 , 0.91]	+
✔ Ferreri	56.2	40.8	105	38.1	53.2	102	8.0%	0.38 [0.11 , 0.66]	+
✔ Gao	22.9	10.79	53	25.47	8.15	53	6.7%	-0.27 [-0.65 , 0.12]	+
✔ Hsih	18	3.54	2	19.1	7.45	41	1.3%	-0.15 [-1.57 , 1.27]	+
✔ Huang	28	12.66	336	19.67	7.49	139	8.9%	0.73 [0.53 , 0.93]	−
✔ Li, Yuanyuan	37	21	31	31	16	23	5.0%	0.31 [-0.23 , 0.85]	+
✔ Mardani	32.1	8.01	70	26.8	8.3	130	7.7%	0.64 [0.35 , 0.94]	−
✔ Meng	40.81	22.62	382	26.26	9.82	383	9.4%	0.83 [0.69 , 0.98]	+
✔ Santotoribio	103.64	88.11	94	72.53	50.19	109	8.0%	0.44 [0.16 , 0.72]	+
✔ Song	24.33	13.61	73	17.4	5.37	231	8.1%	0.85 [0.58 , 1.12]	+
✔ Wu	33.88	15.34	65	28.69	32.66	114	7.6%	0.19 [-0.12 , 0.49]	−
✔ Zhao, Dahai	52.1	69.04	19	23.1	17.99	15	3.8%	0.53 [-0.16 , 1.22]	−
✔ Zhu	31.1	15.8	32	23.7	9.5	84	6.3%	0.64 [0.22 , 1.05]	+
**Total (95% CI)**			**1539**			**1578**	**100.0%**	**0.45 [0.28 , 0.62]**	

Heterogeneity: Tau² = 0.07; Chi² = 57.87, df = 14 (P < 0.00001); I² = 76%
Test for overall effect: Z = 5.14 (P < 0.00001)
Test for subgroup differences: Not applicable

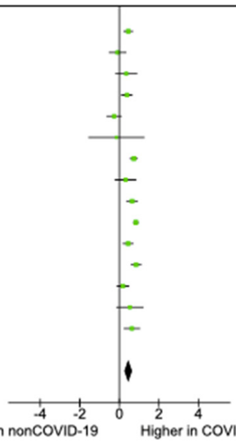

Sensitivity analysis – low risk of bias studies

Study or Subgroup	COVID-19 positive Mean [U/L]	SD [U/L]	Total	COVID-19 negative Mean [U/L]	SD [U/L]	Total	Weight	Std. mean difference IV, Random, 95% CI [U/L]	Risk of Bias A
✔ Brinati	63.61	58.13	175	38.06	53.21	102	12.5%	0.45 [0.20 , 0.70]	+
✖ Chen, Xinyi	28.97	33.87	78	31.78	25.72	26	0.0%	-0.09 [-0.53 , 0.36]	−
✔ Fei	92.63	94.7	24	61.35	80.73	26	7.6%	0.35 [-0.21 , 0.91]	+
✔ Ferreri	56.2	40.8	105	38.1	53.2	102	12.1%	0.38 [0.11 , 0.66]	+
✔ Gao	22.9	10.79	53	25.47	8.15	53	10.3%	-0.27 [-0.65 , 0.12]	+
✔ Hsih	18	3.54	2	19.1	7.45	41	2.1%	-0.15 [-1.57 , 1.27]	+
✖ Huang	28	12.66	336	19.67	7.49	139	0.0%	0.73 [0.53 , 0.93]	−
✔ Li, Yuanyuan	37	21	31	31	16	23	7.8%	0.31 [-0.23 , 0.85]	+
✖ Mardani	32.1	8.01	70	26.8	8.3	130	0.0%	0.64 [0.35 , 0.94]	−
✔ Meng	40.81	22.62	382	26.26	9.82	383	13.9%	0.83 [0.69 , 0.98]	+
✔ Santotoribio	103.64	88.11	94	72.53	50.19	109	12.0%	0.44 [0.16 , 0.72]	+
✔ Song	24.33	13.61	73	17.4	5.37	231	12.1%	0.85 [0.58 , 1.12]	+
✖ Wu	33.88	15.34	65	28.69	32.66	114	0.0%	0.19 [-0.12 , 0.49]	−
✖ Zhao, Dahai	52.1	69.04	19	23.1	17.99	15	0.0%	0.53 [-0.16 , 1.22]	−
✔ Zhu	31.1	15.8	32	23.7	9.5	84	9.7%	0.64 [0.22 , 1.05]	+
**Total (95% CI)**			**971**			**1154**	**100.0%**	**0.46 [0.24 , 0.68]**	

Heterogeneity: $Tau^2 = 0.08$; $Chi^2 = 40.58$, df = 9 ($P < 0.00001$); $I^2 = 78\%$
Test for overall effect: $Z = 4.07$ ($P < 0.0001$)
Test for subgroup differences: Not applicable

Higher in nonCOVID-19 — Higher in COVID-19

Publication bias - funnel plot

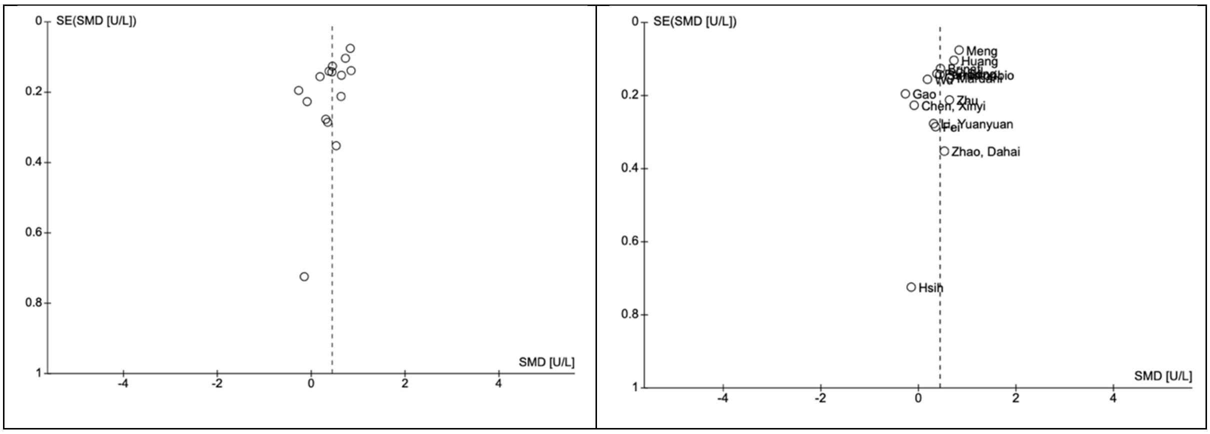

ALT - Alanine aminotransferase, U/L

Study or Subgroup	COVID-19 positive Mean [U/L]	SD [U/L]	Total	COVID-19 negative Mean [U/L]	SD [U/L]	Total	Weight	Std. mean difference IV, Random, 95% CI [U/L]	Risk of Bias A
✔ Brinati	52.16	49.42	164	33.27	35.61	102	6.9%	0.42 [0.17 , 0.67]	+
✔ Chen, Xinyi	28.41	35.72	78	23.53	25.07	26	5.7%	0.14 [-0.30 , 0.59]	−
✔ Fei	40.88	32.52	24	51.65	87.55	26	5.1%	-0.16 [-0.71 , 0.40]	+
✔ Ferreri	47.9	40.9	103	33.1	53.2	102	6.7%	0.31 [0.04 , 0.59]	+
✔ Gao	20.73	12.09	53	24.5	16.84	53	6.1%	-0.26 [-0.64 , 0.13]	+
✔ Hsih	30	42.52	2	16.5	10.37	41	1.8%	1.09 [-0.35 , 2.53]	+
✔ Huang	25.67	17.87	336	23.33	16.48	139	7.1%	0.13 [-0.06 , 0.33]	−
✔ Li, Yuanyuan	45	87	31	26	18	23	5.1%	0.28 [-0.26 , 0.82]	+
✔ Mardani	37.8	7.9	70	26.2	6.9	130	6.4%	1.59 [1.26 , 1.92]	−
✔ Meng	33.85	20.53	382	20.97	9.19	383	7.3%	0.81 [0.66 , 0.96]	+
✔ Peng, Minfei	23.67	13.97	32	17.33	12.82	85	5.9%	0.48 [0.07 , 0.89]	+
✔ Santotoribio	95.56	79.27	94	53.2	37.18	109	6.7%	0.70 [0.41 , 0.98]	+
✔ Song	22.67	12.1	73	13.33	6.71	231	6.7%	1.12 [0.84 , 1.40]	+
✔ Vieceli	37.83	25.73	29	22.33	18.16	71	5.7%	0.75 [0.30 , 1.19]	+
✔ Wu	51.6	39.79	65	33.14	40.05	114	6.5%	0.46 [0.15 , 0.77]	−
✔ Zhao, Dahai	44.39	58.63	19	23.13	17.99	15	4.3%	0.46 [-0.23 , 1.14]	−
✔ Zhu	30.1	31.2	32	23.3	14	84	5.9%	0.33 [-0.07 , 0.74]	+
**Total (95% CI)**			**1587**			**1734**	**100.0%**	**0.50 [0.28 , 0.72]**	

Heterogeneity: $Tau^2 = 0.16$; $Chi^2 = 118.26$, df = 16 ($P < 0.00001$); $I^2 = 86\%$
Test for overall effect: $Z = 4.51$ ($P < 0.00001$)
Test for subgroup differences: Not applicable

Higher in nonCOVID-19 — Higher in COVID-19

Sensitivity analysis – low risk of bias studies

Study or Subgroup	COVID-19 positive Mean [U/L]	SD [U/L]	Total	COVID-19 negative Mean [U/L]	SD [U/L]	Total	Weight	Std. mean difference IV, Random, 95% CI [U/L]	Risk of Bias A
✔ Brinati	52.16	49.42	164	33.27	35.61	102	10.2%	0.42 [0.17 , 0.67]	+
✖ Chen, Xinyi	28.41	35.72	78	23.53	25.07	26	0.0%	0.14 [-0.30 , 0.59]	-
✔ Fei	40.88	32.52	24	51.65	87.55	26	6.8%	-0.16 [-0.71 , 0.40]	+
✔ Ferreri	47.9	40.9	103	33.1	53.2	102	9.9%	0.31 [0.04 , 0.59]	+
✔ Gao	20.73	12.09	53	24.5	16.84	53	8.7%	-0.26 [-0.64 , 0.13]	+
✔ Hsih	30	42.52	2	16.5	10.37	41	2.0%	1.09 [-0.35 , 2.53]	+
✖ Huang	25.67	17.87	336	23.33	16.48	139	0.0%	0.13 [-0.06 , 0.33]	-
✔ Li, Yuanyuan	45	87	31	26	18	23	6.9%	0.28 [-0.26 , 0.82]	+
✖ Mardani	37.8	7.9	70	26.2	6.9	130	0.0%	1.59 [1.26 , 1.92]	-
✔ Meng	33.85	20.53	382	20.97	9.19	383	11.1%	0.81 [0.66 , 0.96]	+
✔ Peng, Minfei	23.67	13.97	32	17.33	12.82	85	8.4%	0.48 [0.07 , 0.89]	+
✔ Santotoribio	95.56	79.27	94	53.2	37.18	109	9.8%	0.70 [0.41 , 0.98]	+
✔ Song	22.67	12.1	73	13.33	6.71	231	9.9%	1.12 [0.84 , 1.40]	+
✔ Vieceli	37.83	25.73	29	22.33	18.16	71	8.0%	0.75 [0.30 , 1.19]	+
✖ Wu	51.6	39.79	65	33.14	40.05	114	0.0%	0.46 [0.15 , 0.77]	-
✖ Zhao, Dahai	44.39	58.63	19	23.13	17.99	15	0.0%	0.46 [-0.23 , 1.14]	-
✔ Zhu	30.1	31.2	32	23.3	14	84	8.4%	0.33 [-0.07 , 0.74]	+
**Total (95% CI)**			**1019**			**1310**	**100.0%**	**0.48 [0.25 , 0.70]**	

Heterogeneity: Tau² = 0.11; Chi² = 58.34, df = 11 (P < 0.00001); I² = 81%
Test for overall effect: Z = 4.16 (P < 0.0001)
Test for subgroup differences: Not applicable

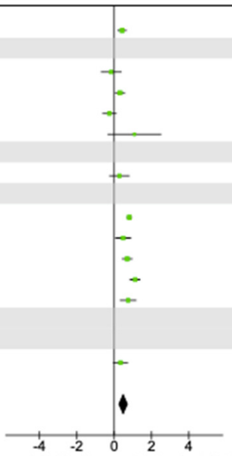

Publication bias - funnel plot

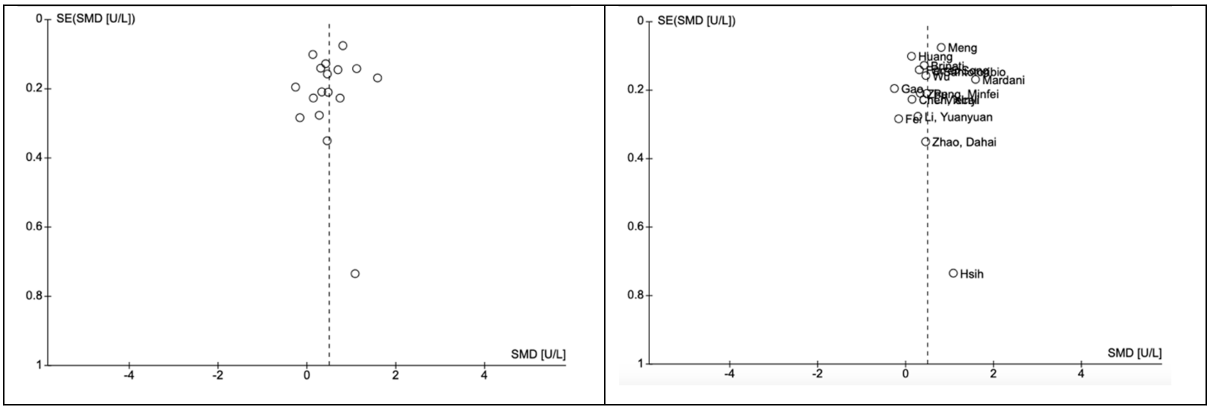

Total bilirubin, umol/L

Study or Subgroup	COVID-19 positive Mean [umol/L]	SD [umol/L]	Total	COVID-19 negative Mean [umol/L]	SD [umol/L]	Total	Weight	Std. mean difference IV, Random, 95% CI [umol/L]	Risk of Bias A
✔ Chen, Xinyi	9.91	6.63	78	14.35	7.32	26	9.5%	-0.65 [-1.10 , -0.19]	-
✔ Gao	10.51	4.14	53	9.67	4.95	53	10.2%	0.18 [-0.20 , 0.56]	+
✔ Hsih	6.16	0.61	2	9.52	3.81	41	2.8%	-0.88 [-2.31 , 0.56]	+
✔ Huang	9.9	5.58	336	11.93	6.22	139	11.9%	-0.35 [-0.55 , -0.15]	-
✔ Li, Yuanyuan	12	7	31	11	6	23	8.5%	0.15 [-0.39 , 0.69]	+
✔ Martín-Sánchez	8.98	0.86	1190	9.16	3.03	803	12.6%	-0.09 [-0.18 , 0.00]	+
✔ Meng	12.32	3.87	382	10.37	3.97	383	12.3%	0.50 [0.35 , 0.64]	+
✔ Peng, Minfei	3.4	1.86	32	4.2	2.34	85	9.9%	-0.36 [-0.77 , 0.05]	+
✔ Santotoribio	9.67	3.91	94	17.47	15.54	109	11.2%	-0.66 [-0.95 , -0.38]	+
✔ Wu	30.1	31.81	65	17.25	20.07	114	11.0%	0.51 [0.20 , 0.82]	-
**Total (95% CI)**			**2263**			**1776**	**100.0%**	**-0.10 [-0.37 , 0.17]**	

Heterogeneity: Tau² = 0.15; Chi² = 106.44, df = 9 (P < 0.00001); I² = 92%
Test for overall effect: Z = 0.72 (P = 0.47)
Test for subgroup differences: Not applicable

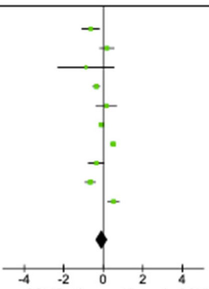

Sensitivity analysis – low risk of bias studies

Study or Subgroup	COVID-19 positive Mean [umol/L]	SD [umol/L]	Total	COVID-19 negative Mean [umol/L]	SD [umol/L]	Total	Weight	Std. mean difference IV, Random, 95% CI [umol/L]	Risk of Bias A
✖ Chen, Xinyi	9.91	6.63	78	14.35	7.32	26	0.0%	-0.65 [-1.10 , -0.19]	−
✔ Gao	10.51	4.14	53	9.67	4.95	53	15.1%	0.18 [-0.20 , 0.56]	+
✔ Hsih	6.16	0.61	2	9.52	3.81	41	4.3%	-0.88 [-2.31 , 0.56]	+
✖ Huang	9.9	5.58	336	11.93	6.22	139	0.0%	-0.35 [-0.55 , -0.15]	−
✔ Li, Yuanyuan	12	7	31	11	6	23	12.7%	0.15 [-0.39 , 0.69]	+
✔ Martín-Sánchez	8.98	0.86	1190	9.16	3.03	803	18.4%	-0.09 [-0.18 , 0.00]	+
✔ Meng	12.32	3.87	382	10.37	3.97	383	18.1%	0.50 [0.35 , 0.64]	+
✔ Peng, Minfei	3.4	1.86	32	4.2	2.34	85	14.7%	-0.36 [-0.77 , 0.05]	+
✔ Santotoribio	9.67	3.91	94	17.47	15.54	109	16.5%	-0.66 [-0.95 , -0.38]	+
✖ Wu	30.1	31.81	65	17.25	20.07	114	0.0%	0.51 [0.20 , 0.82]	−
**Total (95% CI)**			**1784**			**1497**	**100.0%**	**-0.08 [-0.42 , 0.26]**	

Heterogeneity: Tau² = 0.16; Chi² = 75.90, df = 6 (P < 0.00001); I² = 92%
Test for overall effect: Z = 0.46 (P = 0.64)
Test for subgroup differences: Not applicable

Std. mean difference IV, Random, 95% CI [umol/L]: -4 -2 0 2 4 — Higher in nonCOVID-19 / Higher in COVID-19

Publication bias - funnel plot

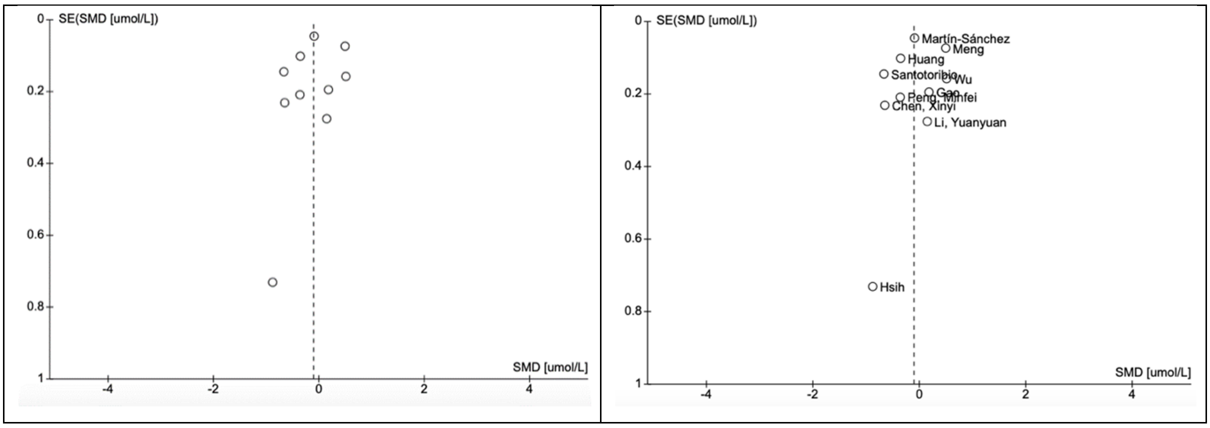

Albumin, g/L

Study or Subgroup	COVID-19 positive Mean [g/L]	SD [g/L]	Total	COVID-19 negative Mean [g/L]	SD [g/L]	Total	Weight	Std. mean difference IV, Random, 95% CI [g/L]	Risk of Bias A
✔ Gao	42.18	6.53	53	44.27	5.11	53	10.9%	-0.35 [-0.74 , 0.03]	+
✔ Huang	42.67	5.14	336	41.77	5.54	139	12.5%	0.17 [-0.03 , 0.37]	−
✔ Li, Yuanyuan	40	5	31	38	5	23	9.2%	0.39 [-0.15 , 0.94]	+
✔ Mardani	29	8	70	37	8	130	11.6%	-1.00 [-1.30 , -0.69]	−
✔ Meng	34.34	3.6	382	36.49	4.01	383	12.9%	-0.56 [-0.71 , -0.42]	+
✔ Nalbant	34	5	54	34	6	26	10.0%	0.00 [-0.47 , 0.47]	+
✔ Peng, Minfei	41	4.66	32	40.67	5.28	85	10.7%	0.06 [-0.34 , 0.47]	+
✔ Wu	35.97	5.53	65	39.74	4.93	114	11.6%	-0.73 [-1.04 , -0.41]	−
✔ Zhao, Guolian	40	4.4	47	41.7	3.8	45	10.6%	-0.41 [-0.82 , 0.00]	−
**Total (95% CI)**			**1070**			**998**	**100.0%**	**-0.29 [-0.59 , 0.01]**	

Heterogeneity: Tau² = 0.18; Chi² = 70.91, df = 8 (P < 0.00001); I² = 89%
Test for overall effect: Z = 1.90 (P = 0.06)
Test for subgroup differences: Not applicable

Std. mean difference IV, Random, 95% CI [g/L]: -4 -2 0 2 4 — Higher in nonCOVID-19 / Higher in COVID-19

Sensitivity analysis – low risk of bias studies

Study or Subgroup	COVID-19 positive Mean [g/L]	SD [g/L]	Total	COVID-19 negative Mean [g/L]	SD [g/L]	Total	Weight	Std. mean difference IV, Random, 95% CI [g/L]	Risk of Bias A
✔ Gao	42.18	6.53	53	44.27	5.11	53	20.3%	-0.35 [-0.74 , 0.03]	+
✖ Huang	42.67	5.14	336	41.77	5.54	139	0.0%	0.17 [-0.03 , 0.37]	-
✔ Li, Yuanyuan	40	5	31	38	5	23	16.5%	0.39 [-0.15 , 0.94]	+
✖ Mardani	29	8	70	37	8	130	0.0%	-1.00 [-1.30 , -0.69]	-
✔ Meng	34.34	3.6	382	36.49	4.01	383	25.2%	-0.56 [-0.71 , -0.42]	+
✔ Nalbant	34	5	54	34	6	26	18.3%	0.00 [-0.47 , 0.47]	+
✔ Peng, Minfei	41	4.66	32	40.67	5.28	85	19.7%	0.06 [-0.34 , 0.47]	+
✖ Wu	35.97	5.53	65	39.74	4.93	114	0.0%	-0.73 [-1.04 , -0.41]	-
✖ Zhao, Guolian	40	4.4	47	41.7	3.8	45	0.0%	-0.41 [-0.82 , 0.00]	-
**Total (95% CI)**			**552**			**570**	**100.0%**	**-0.14 [-0.50 , 0.23]**	

Heterogeneity: Tau² = 0.13; Chi² = 20.95, df = 4 (P = 0.0003); I² = 81%
Test for overall effect: Z = 0.73 (P = 0.46)
Test for subgroup differences: Not applicable

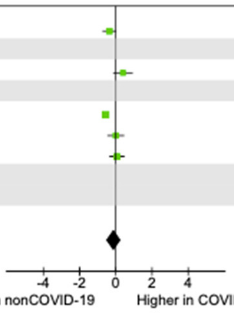

Publication bias - funnel plot

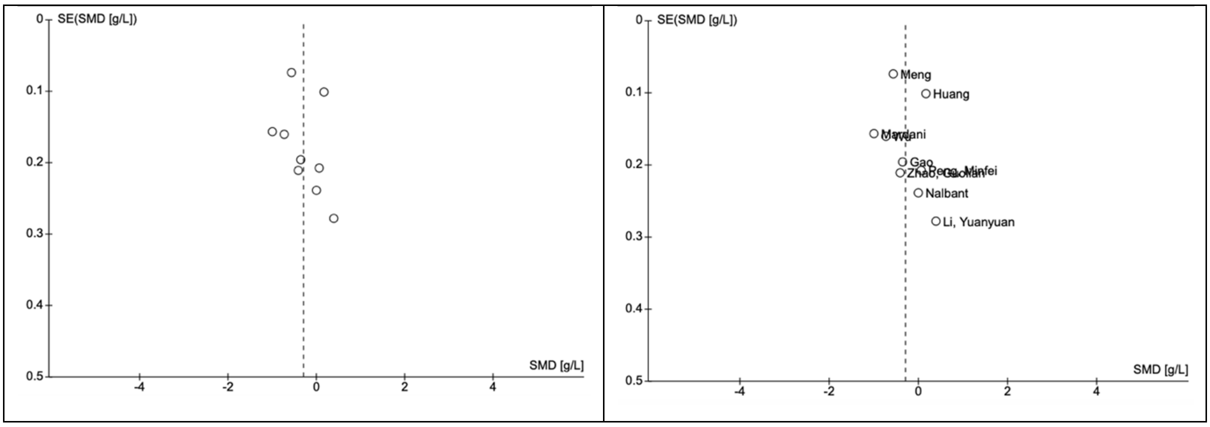

Procalcitonin, ng/mL

Study or Subgroup	COVID-19 positive Mean [ng/mL]	SD [ng/mL]	Total	COVID-19 negative Mean [ng/mL]	SD [ng/mL]	Total	Weight	Std. mean difference IV, Random, 95% CI [ng/mL]	Risk of Bias A
✔ Chen, Xiaofeng	0.26	0.84	70	3.36	8.98	66	10.1%	-0.49 [-0.83 , -0.15]	+
✔ Chen, Xinyi	0.1	1.24	78	1.97	2.61	26	9.9%	-1.10 [-1.57 , -0.63]	-
✔ Fei	3.34	9.74	24	5.9	20.17	26	9.8%	-0.16 [-0.71 , 0.40]	+
✔ Huang	0.07	0.06	336	0.08	0.07	139	10.3%	-0.16 [-0.36 , 0.04]	-
✔ Li, Yuanyuan	0.11	0.12	31	0.07	0.08	23	9.8%	0.38 [-0.17 , 0.92]	+
✔ Martín-Sánchez	0.1	0.03	1190	0.06	0.03	803	10.3%	1.33 [1.23 , 1.43]	+
✔ Peng, Minfei	0.04	0.03	32	0.08	0.1	85	10.0%	-0.46 [-0.87 , -0.05]	+
✔ Pisapia	0.03	0.1	17	0.1	0.1	20	9.5%	-0.68 [-1.35 , -0.02]	+
✔ Song	0.08	0.11	91	0.18	0.13	308	10.2%	-0.79 [-1.03 , -0.55]	+
✔ Zhu	0.1	0.1	32	0.2	0.2	84	10.0%	-0.56 [-0.97 , -0.14]	+
**Total (95% CI)**			**1901**			**1580**	**100.0%**	**-0.26 [-0.97 , 0.45]**	

Heterogeneity: Tau² = 1.27; Chi² = 564.56, df = 9 (P < 0.00001); I² = 98%
Test for overall effect: Z = 0.73 (P = 0.47)
Test for subgroup differences: Not applicable

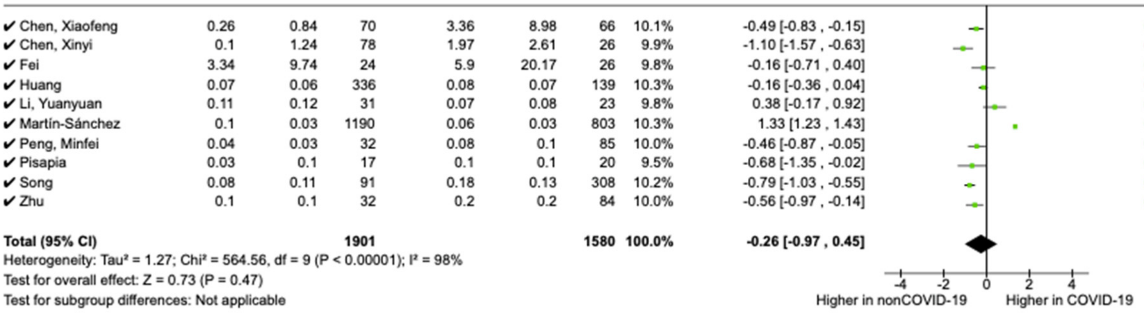

Sensitivity analysis – low risk of bias studies

Study or Subgroup	COVID-19 positive Mean [ng/mL]	SD [ng/mL]	Total	COVID-19 negative Mean [ng/mL]	SD [ng/mL]	Total	Weight	Std. mean difference IV, Random, 95% CI [ng/mL]	Risk of Bias A
✔ Chen, Xiaofeng	0.26	0.84	70	3.36	8.98	66	12.7%	-0.49 [-0.83 , -0.15]	+
✖ Chen, Xinyi	0.1	1.24	78	1.97	2.61	26	0.0%	-1.10 [-1.57 , -0.63]	−
✔ Fei	3.34	9.74	24	5.9	20.17	26	12.3%	-0.16 [-0.71 , 0.40]	+
✖ Huang	0.07	0.06	336	0.08	0.07	139	0.0%	-0.16 [-0.36 , 0.04]	−
✔ Li, Yuanyuan	0.11	0.12	31	0.07	0.08	23	12.3%	0.38 [-0.17 , 0.92]	+
✔ Martín-Sánchez	0.1	0.03	1190	0.06	0.03	803	12.9%	1.33 [1.23 , 1.43]	+
✔ Peng, Minfei	0.04	0.03	32	0.08	0.1	85	12.6%	-0.46 [-0.87 , -0.05]	+
✔ Pisapia	0.03	0.1	17	0.1	0.1	20	12.0%	-0.68 [-1.35 , -0.02]	+
✔ Song	0.08	0.11	91	0.18	0.13	308	12.8%	-0.79 [-1.03 , -0.55]	+
✔ Zhu	0.1	0.1	32	0.2	0.2	84	12.5%	-0.56 [-0.97 , -0.14]	+
**Total (95% CI)**			**1487**			**1415**	**100.0%**	**-0.17 [-1.04 , 0.69]**	

Heterogeneity: Tau² = 1.51; Chi² = 444.72, df = 7 (P < 0.00001); I² = 98%
Test for overall effect: Z = 0.39 (P = 0.69)
Test for subgroup differences: Not applicable

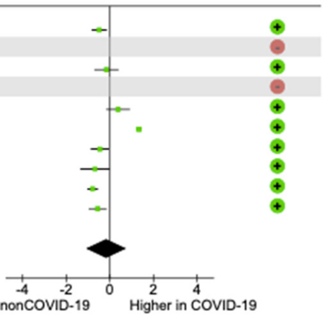

Publication bias - funnel plot

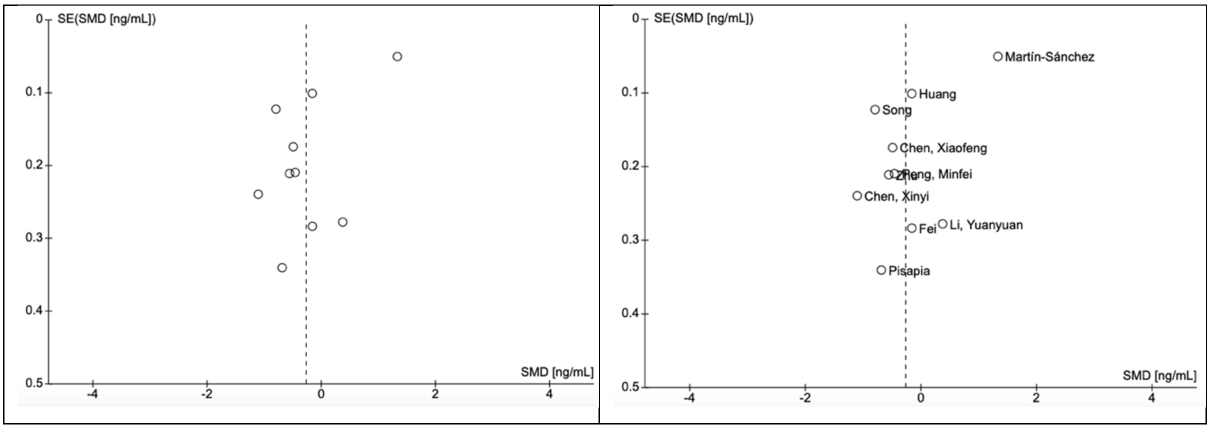

C-Reactive Protein, mg/dL

Study or Subgroup	COVID-19 positive Mean [mg/dL]	SD [mg/dL]	Total	COVID-19 negative Mean [mg/dL]	SD [mg/dL]	Total	Weight	Std. mean difference IV, Random, 95% CI [mg/dL]	Risk of Bias A
✔ Brinati	107.23	98.64	177	63.05	79.81	101	4.1%	0.48 [0.23 , 0.73]	+
✔ Chen, Xiaofeng	26.37	30.97	70	69.3	65.88	66	3.9%	-0.84 [-1.19 , -0.49]	+
✔ Chen, Xinyi	30.97	40.72	78	49.54	49.74	26	3.7%	-0.43 [-0.88 , 0.02]	−
✔ Cheng	16.83	15.44	11	33.43	65.36	22	3.0%	-0.30 [-1.02 , 0.43]	+
✔ Chi	29.27	31.3	17	17.25	23.31	51	3.4%	0.47 [-0.09 , 1.02]	−
✔ de Moraes Batista	45.2	50.1	102	33	56.2	133	4.1%	0.23 [-0.03 , 0.49]	+
✔ Fei	178.01	99.52	0	90.84	100.34	26		Not estimable	+
✔ Feng	0.87	0.98	33	0.48	0.82	19	3.4%	0.42 [-0.16 , 0.99]	+
✔ Ferreri	87.1	81.2	105	63.1	79.8	101	4.1%	0.30 [0.02 , 0.57]	+
✔ Gao	9.89	20.3	53	16.23	28.27	53	3.9%	-0.26 [-0.64 , 0.13]	+
✔ Hsih	2.3	1.06	2	6.5	10.52	41	1.6%	-0.40 [-1.82 , 1.03]	+
✔ Huang	17.3	18.69	336	28.67	42.63	139	4.2%	-0.41 [-0.61 , -0.21]	−
✔ Kurstjens	106.59	70.78	669	79.99	96.88	298	4.3%	0.33 [0.20 , 0.47]	+
✔ Li, Qilin	34.37	43.06	458	6.13	10.63	531	4.3%	0.93 [0.80 , 1.06]	−
✔ Li, Yuanyuan	41	39	31	36	34	23	3.5%	0.13 [-0.41 , 0.67]	+
✔ Martín-Sánchez	62.32	25.46	1190	40.19	24.26	803	4.3%	0.89 [0.79 , 0.98]	+
✔ O'Reilly	67	99	11	46	75	229	3.3%	0.27 [-0.33 , 0.88]	+
✔ Peng, Ling	7.66	7.33	11	10	13.75	75	3.2%	-0.18 [-0.81 , 0.46]	+
✔ Peng, Minfei	12.5	14.59	32	14.63	19.31	85	3.8%	-0.12 [-0.52 , 0.29]	+
✔ Pisapia	3.4	4.8	17	2.8	4.2	20	3.2%	0.13 [-0.52 , 0.78]	+
✔ Qin	11.7	15.3	88	16.27	23.03	80	4.0%	-0.23 [-0.54 , 0.07]	−
✔ Song	29.52	30.92	91	6.47	12.29	308	4.1%	1.26 [1.01 , 1.51]	+
✔ Tordjman	58.8	45.9	50	64.6	116.4	50	3.8%	-0.07 [-0.46 , 0.33]	+
✔ Vieceli	92	74.08	29	75.6	105.41	71	3.7%	0.17 [-0.27 , 0.60]	+
✔ Wang	11.79	15.86	89	22.91	41.58	89	4.0%	-0.35 [-0.65 , -0.06]	+
✔ Wu	33.64	47.42	65	40.56	47.71	114	4.0%	-0.14 [-0.45 , 0.16]	−
✔ Zhao, Dahai	54.52	93.79	19	54.69	115.98	15	3.1%	-0.00 [-0.68 , 0.68]	−
✔ Zhu	20.7	24	32	18.7	28.2	84	3.8%	0.07 [-0.33 , 0.48]	+
**Total (95% CI)**			**3866**			**3653**	**100.0%**	**0.11 [-0.11 , 0.33]**	

Heterogeneity: Tau² = 0.29; Chi² = 426.80, df = 26 (P < 0.00001); I² = 94%
Test for overall effect: Z = 0.97 (P = 0.33)
Test for subgroup differences: Not applicable

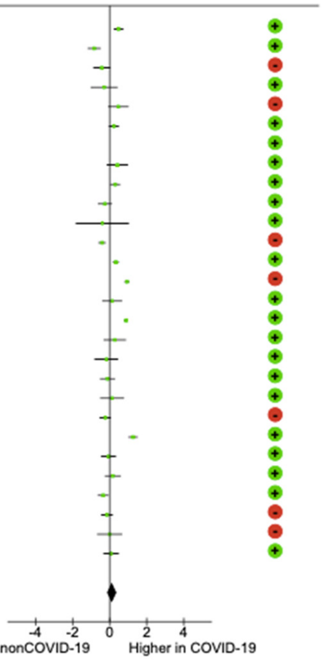

Sensitivity analysis – low risk of bias studies

Study or Subgroup	COVID-19 positive Mean [mg/dL]	SD [mg/dL]	Total	COVID-19 negative Mean [mg/dL]	SD [mg/dL]	Total	Weight	Std. mean difference IV, Random, 95% CI [mg/dL]	Risk of Bias A
✔ Brinati	107.23	98.64	177	63.05	79.81	101	5.7%	0.48 [0.23 , 0.73]	+
✔ Chen, Xiaofeng	26.37	30.97	70	69.3	65.88	66	5.4%	-0.84 [-1.19 , -0.49]	+
✖ Chen, Xinyi	30.97	40.72	78	49.54	49.74	26	0.0%	-0.43 [-0.88 , 0.02]	−
✔ Cheng	16.83	15.44	11	33.43	65.36	22	4.0%	-0.30 [-1.02 , 0.43]	+
✖ Chi	29.27	31.3	17	17.25	23.31	51	0.0%	0.47 [-0.09 , 1.02]	−
✔ de Moraes Batista	45.2	50.1	102	33	56.2	133	5.7%	0.23 [-0.03 , 0.49]	+
✔ Fei	178.01	99.52	0	90.84	100.34	26		Not estimable	+
✔ Feng	0.87	0.98	33	0.48	0.82	19	4.6%	0.42 [-0.16 , 0.99]	+
✔ Ferreri	87.1	81.2	105	63.1	79.8	101	5.6%	0.30 [0.02 , 0.57]	+
✔ Gao	9.89	20.3	53	16.23	28.27	53	5.3%	-0.26 [-0.64 , 0.13]	+
✔ Hsih	2.3	1.06	2	6.5	10.52	41	2.0%	-0.40 [-1.82 , 1.03]	+
✖ Huang	17.3	18.69	336	28.67	42.63	139	0.0%	-0.41 [-0.61 , -0.21]	−
✔ Kurstjens	106.59	70.78	669	79.99	96.88	298	6.0%	0.33 [0.20 , 0.47]	+
✖ Li, Qilin	34.37	43.06	458	6.13	10.63	531	0.0%	0.93 [0.80 , 1.06]	−
✔ Li, Yuanyuan	41	39	31	36	34	23	4.7%	0.13 [-0.41 , 0.67]	+
✔ Martín-Sánchez	62.32	25.46	1190	40.19	24.26	803	6.0%	0.89 [0.79 , 0.98]	+
✔ O`Reilly	67	99	11	46	75	229	4.4%	0.27 [-0.33 , 0.88]	+
✔ Peng, Ling	7.66	7.33	11	10	13.75	75	4.3%	-0.18 [-0.81 , 0.46]	+
✔ Peng, Minfei	12.5	14.59	32	14.63	19.31	85	5.2%	-0.12 [-0.52 , 0.29]	+
✔ Pisapia	3.4	4.8	17	2.8	4.2	20	4.3%	0.13 [-0.52 , 0.78]	+
✖ Qin	11.7	15.3	88	16.27	23.03	80	0.0%	-0.23 [-0.54 , 0.07]	−
✔ Song	29.52	30.92	91	6.47	12.29	308	5.7%	1.26 [1.01 , 1.51]	+
✔ Tordjman	58.8	45.9	50	64.6	116.4	50	5.3%	-0.07 [-0.46 , 0.33]	+
✔ Vieceli	92	74.08	29	75.6	105.41	71	5.1%	0.17 [-0.27 , 0.60]	+
✔ Wang	11.79	15.86	89	22.91	41.58	89	5.6%	-0.35 [-0.65 , -0.06]	+
✖ Wu	33.64	47.42	65	40.56	47.71	114	0.0%	-0.14 [-0.45 , 0.16]	−
✖ Zhao, Dahai	54.52	93.79	19	54.69	115.98	15	0.0%	-0.00 [-0.68 , 0.68]	−
✔ Zhu	20.7	24	32	18.7	28.2	84	5.2%	0.07 [-0.33 , 0.48]	+
**Total (95% CI)**			**2805**			**2697**	**100.0%**	**0.14 [-0.10 , 0.39]**	

Heterogeneity: $Tau^2 = 0.26$; $Chi^2 = 252.46$, df = 19 ($P < 0.00001$); $I^2 = 92\%$
Test for overall effect: Z = 1.14 (P = 0.26)
Test for subgroup differences: Not applicable

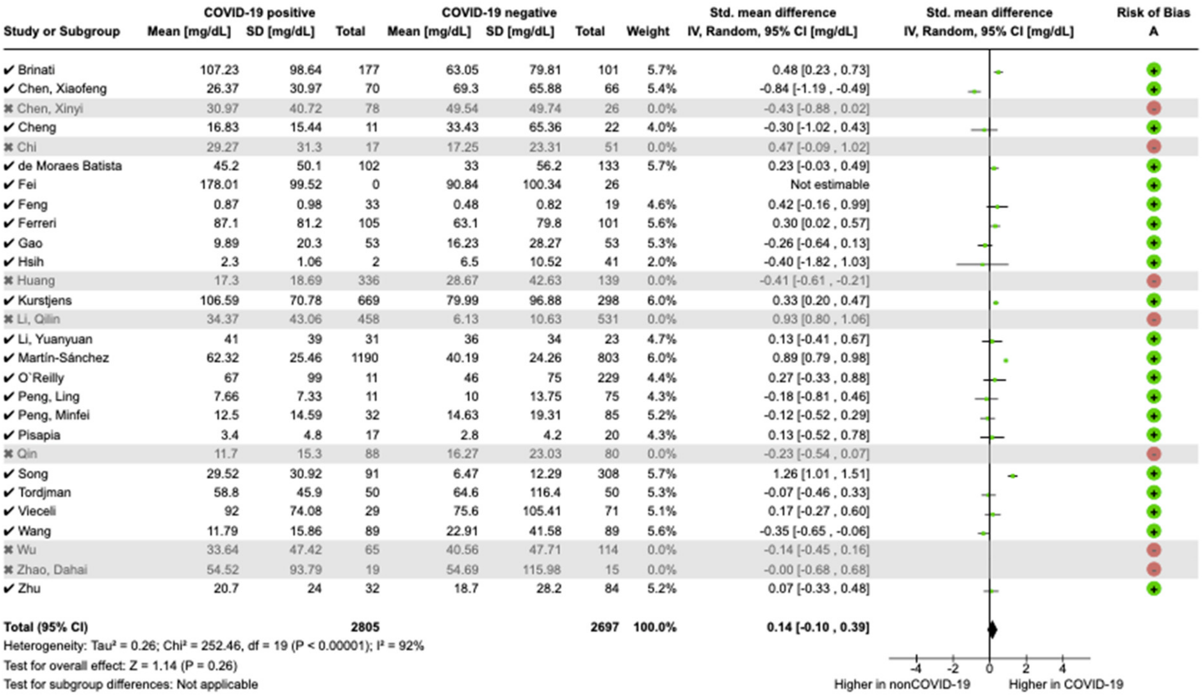

Publication bias - funnel plot

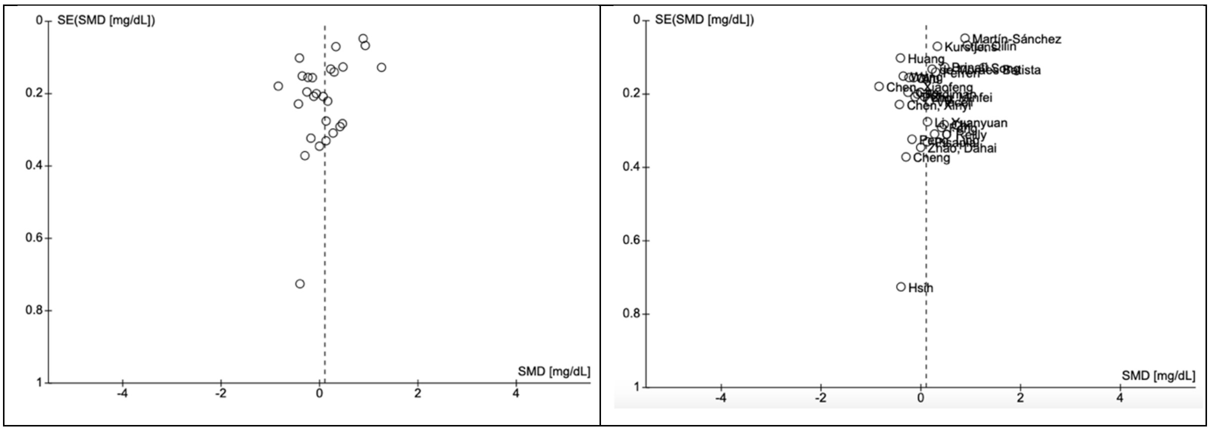

Urea BUN, mg/dL

Study or Subgroup	COVID-19 positive Mean	SD	Total	COVID-19 negative Mean	SD	Total	Weight	Std. mean difference IV, Random, 95% CI	Risk of Bias A
✔ Chen, Xinyi	3.96	2.82	78	6.47	2.67	26	19.5%	-0.89 [-1.36 , -0.43]	−
✔ Fei	9.55	6.21	24	10.89	6.85	26	18.8%	-0.20 [-0.76 , 0.35]	+
✔ Huang	3.93	1.3	336	4.27	1.65	139	20.8%	-0.24 [-0.44 , -0.04]	−
✔ Mardani	5.76	1.43	70	4.3	0.97	130	20.3%	1.26 [0.95 , 1.58]	−
✔ Song	5.37	2.34	73	4.23	1.21	231	20.6%	0.73 [0.46 , 1.00]	+
**Total (95% CI)**			**581**			**552**	**100.0%**	**0.15 [-0.57 , 0.86]**	

Heterogeneity: $Tau^2 = 0.63$; $Chi^2 = 100.95$, df = 4 ($P < 0.00001$); $I^2 = 96\%$
Test for overall effect: Z = 0.40 (P = 0.69)
Test for subgroup differences: Not applicable

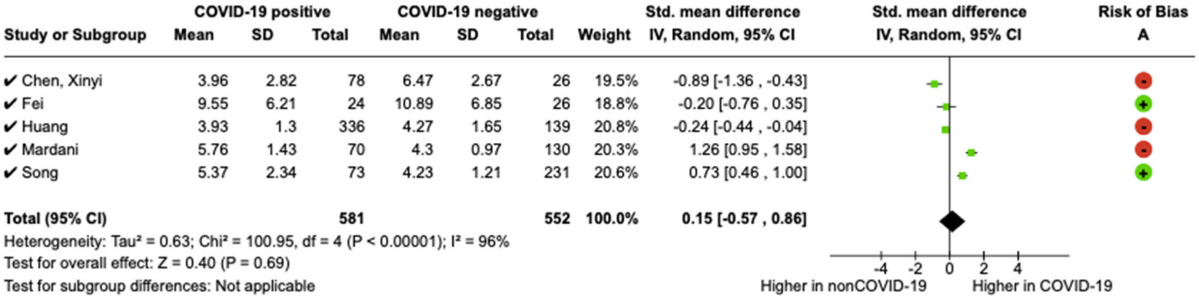

Sensitivity analysis – low risk of bias studies

Study or Subgroup	COVID-19 positive Mean	SD	Total	COVID-19 negative Mean	SD	Total	Weight	Std. mean difference IV, Random, 95% CI	Risk of Bias A
✖ Chen, Xinyi	3.96	2.82	78	6.47	2.67	26	0.0%	-0.89 [-1.36 , -0.43]	−
✔ Fei	9.55	6.21	24	10.89	6.85	26	46.5%	-0.20 [-0.76 , 0.35]	+
✖ Huang	3.93	1.3	336	4.27	1.65	139	0.0%	-0.24 [-0.44 , -0.04]	−
✖ Mardani	5.76	1.43	70	4.3	0.97	130	0.0%	1.26 [0.95 , 1.58]	−
✔ Song	5.37	2.34	73	4.23	1.21	231	53.5%	0.73 [0.46 , 1.00]	+
**Total (95% CI)**			**97**			**257**	**100.0%**	**0.30 [-0.61 , 1.21]**	

Heterogeneity: Tau² = 0.38; Chi² = 8.74, df = 1 (P = 0.003); I² = 89%
Test for overall effect: Z = 0.64 (P = 0.52)
Test for subgroup differences: Not applicable

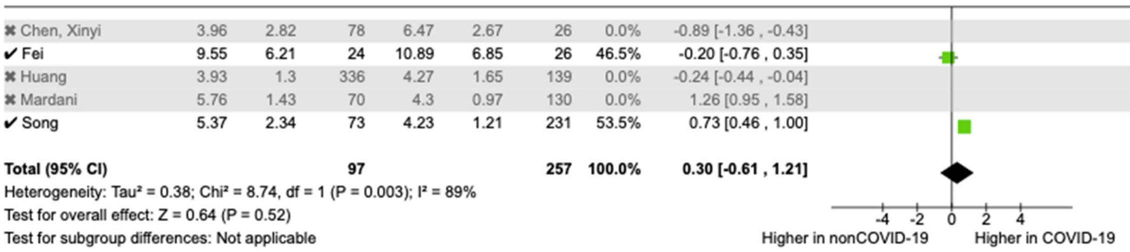

Publication bias - funnel plot

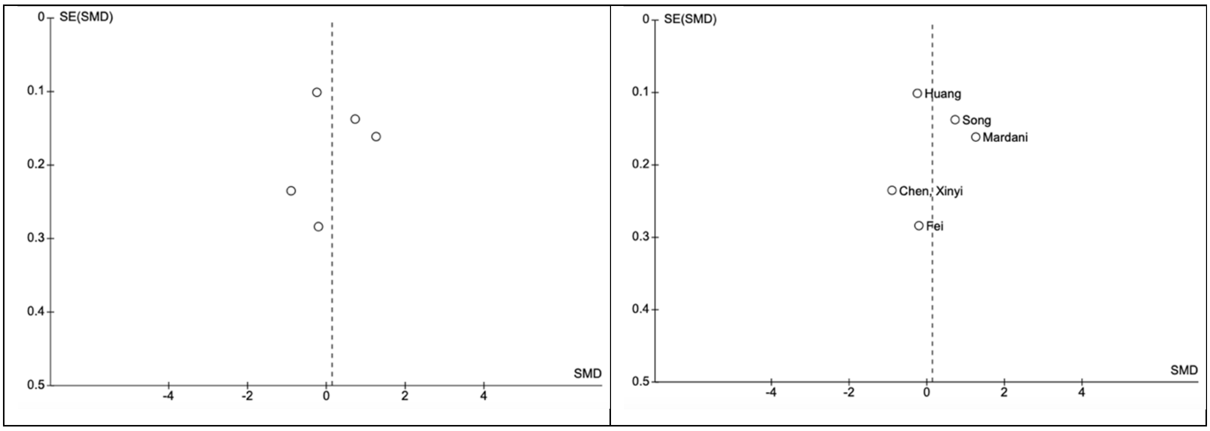

D - dimer, mg/L

Study or Subgroup	COVID-19 positive Mean [mg/L]	SD [mg/L]	Total	COVID-19 negative Mean [mg/L]	SD [mg/L]	Total	Weight	Std. mean difference IV, Random, 95% CI [mg/L]	Risk of Bias A
✔ Fei	5.52	7.07	24	3.57	4.86	26	9.3%	0.32 [-0.24 , 0.88]	+
✔ Gao	0.4	0.31	53	0.42	0.31	53	11.1%	-0.06 [-0.44 , 0.32]	+
✔ Han	10.36	25.31	94	0.26	0.18	40	11.1%	0.47 [0.10 , 0.85]	−
✔ Huang	0.81	1.17	336	0.44	0.52	139	12.5%	0.36 [0.16 , 0.56]	−
✔ Li, Yuanyuan	0.5	0.5	31	0.8	1.2	23	9.5%	-0.34 [-0.88 , 0.20]	+
✔ Martín-Sánchez	0.69	0.12	1190	0.54	0.25	803	13.0%	0.82 [0.72 , 0.91]	+
✔ Peng, Minfei	0.28	0.15	32	0.38	0.35	85	10.8%	-0.32 [-0.73 , 0.09]	+
✔ Santotoribio	15.37	20.31	94	3.17	3.68	109	11.9%	0.86 [0.57 , 1.15]	+
✔ Zhu	0.2	0.2	32	0.3	0.5	84	10.8%	-0.23 [-0.63 , 0.18]	+
**Total (95% CI)**			**1886**			**1362**	**100.0%**	**0.24 [-0.08 , 0.55]**	

Heterogeneity: Tau² = 0.20; Chi² = 89.22, df = 8 (P < 0.00001); I² = 91%
Test for overall effect: Z = 1.48 (P = 0.14)
Test for subgroup differences: Not applicable

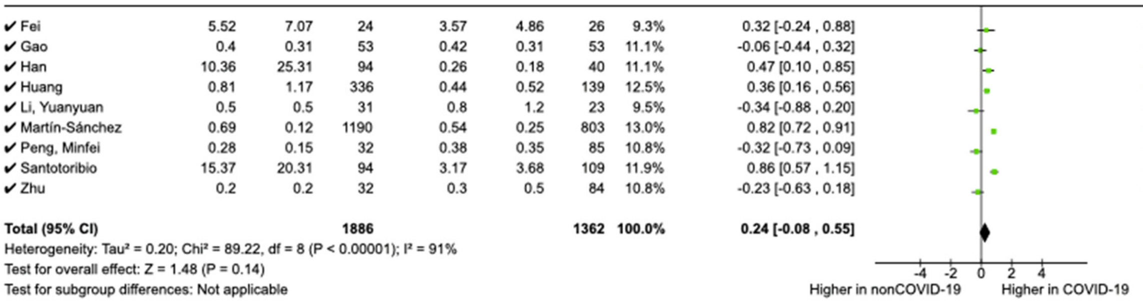

Sensitivity analysis – low risk of bias studies

Study or Subgroup	COVID-19 positive Mean [mg/L]	SD [mg/L]	Total	COVID-19 negative Mean [mg/L]	SD [mg/L]	Total	Weight	Std. mean difference IV, Random, 95% CI [mg/L]	Risk of Bias A
✔ Fei	5.52	7.07	24	3.57	4.86	26	12.8%	0.32 [-0.24 , 0.88]	+
✔ Gao	0.4	0.31	53	0.42	0.31	53	14.5%	-0.06 [-0.44 , 0.32]	+
✖ Han	10.36	25.31	94	0.26	0.18	40	0.0%	0.47 [0.10 , 0.85]	−
✖ Huang	0.81	1.17	336	0.44	0.52	139	0.0%	0.36 [0.16 , 0.56]	−
✔ Li, Yuanyuan	0.5	0.5	31	0.8	1.2	23	13.0%	-0.34 [-0.88 , 0.20]	+
✔ Martín-Sánchez	0.69	0.12	1190	0.54	0.25	803	16.1%	0.82 [0.72 , 0.91]	+
✔ Peng, Minfei	0.28	0.15	32	0.38	0.35	85	14.2%	-0.32 [-0.73 , 0.09]	+
✔ Santotoribio	15.37	20.31	94	3.17	3.68	109	15.2%	0.86 [0.57 , 1.15]	+
✔ Zhu	0.2	0.2	32	0.3	0.5	84	14.2%	-0.23 [-0.63 , 0.18]	+
**Total (95% CI)**			**1456**			**1183**	**100.0%**	**0.17 [-0.27 , 0.61]**	

Heterogeneity: Tau² = 0.31; Chi² = 81.29, df = 6 (P < 0.00001); I² = 93%
Test for overall effect: Z = 0.77 (P = 0.44)
Test for subgroup differences: Not applicable

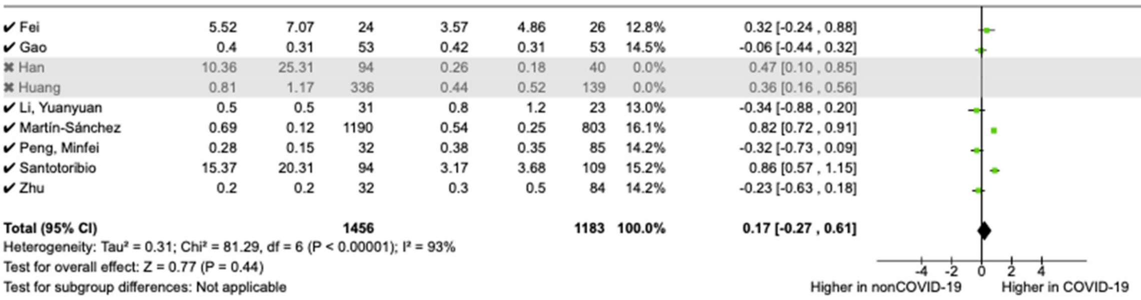

Publication bias - funnel plot

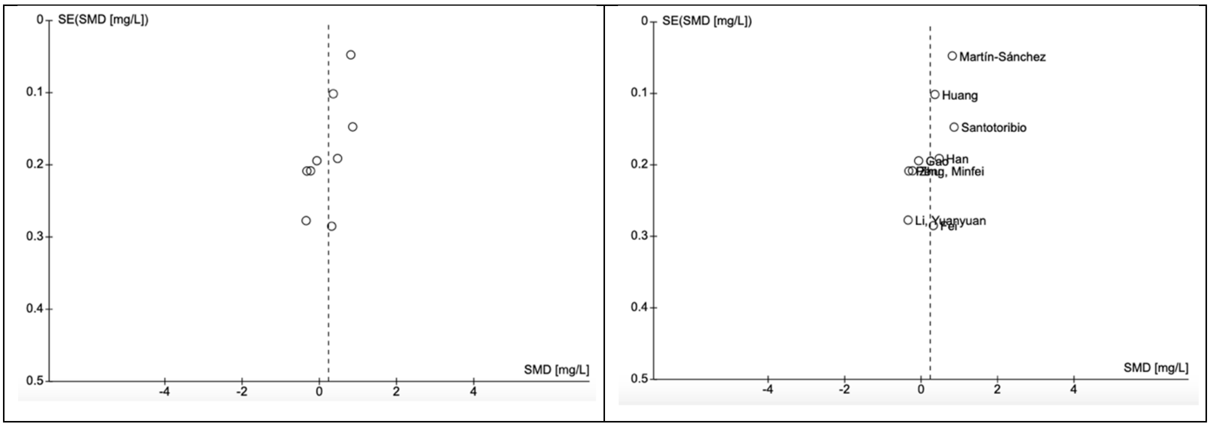

Creatinine, umol/L

Study or Subgroup	COVID-19 positive Mean [umol/L]	SD [umol/L]	Total	COVID-19 negative Mean [umol/L]	SD [umol/L]	Total	Weight	Std. mean difference IV, Random, 95% CI [umol/L]	Risk of Bias A
✔ Chen, Xinyi	62.47	38.25	78	78.62	37.85	26	7.0%	-0.42 [-0.87 , 0.03]	-
✔ Fei	123.76	61.88	24	183.88	171.5	26	6.3%	-0.45 [-1.01 , 0.11]	+
✔ Gao	64.98	19.37	53	65.73	18.59	53	7.5%	-0.04 [-0.42 , 0.34]	+
✔ Hsih	71.33	51.68	2	61.29	13.58	41	2.4%	0.63 [-0.80 , 2.06]	+
✔ Huang	65.23	18.76	336	76	20.23	139	8.5%	-0.56 [-0.76 , -0.36]	-
✔ Li, Yuanyuan	69	22	31	62	16	23	6.4%	0.35 [-0.19 , 0.89]	+
✔ Martín-Sánchez	79.66	27.57	1190	90.38	26.24	803	8.8%	-0.40 [-0.49 , -0.31]	+
✔ Peng, Minfei	77.33	19.4	32	71	19.61	85	7.3%	0.32 [-0.09 , 0.73]	+
✔ Santotoribio	167.7	119.11	94	104.81	40.87	109	8.0%	0.73 [0.44 , 1.01]	+
✔ Song	77.83	23.07	73	67.33	17.9	231	8.1%	0.54 [0.28 , 0.81]	+
✔ Tordjman	95.6	65.8	50	84	28.1	50	7.4%	0.23 [-0.17 , 0.62]	+
✔ Vieceli	85.45	25.5	29	90.17	38.8	71	7.1%	-0.13 [-0.56 , 0.30]	+
✔ Wu	56.42	23.48	65	84.62	63.48	114	7.9%	-0.53 [-0.84 , -0.22]	-
✔ Zhu	70.7	19.6	32	65.7	17.1	84	7.3%	0.28 [-0.13 , 0.69]	+
**Total (95% CI)**			**2089**			**1855**	**100.0%**	**0.01 [-0.25 , 0.26]**	

Heterogeneity: Tau² = 0.19; Chi² = 128.82, df = 13 (P < 0.00001); I² = 90%
Test for overall effect: Z = 0.05 (P = 0.96)
Test for subgroup differences: Not applicable

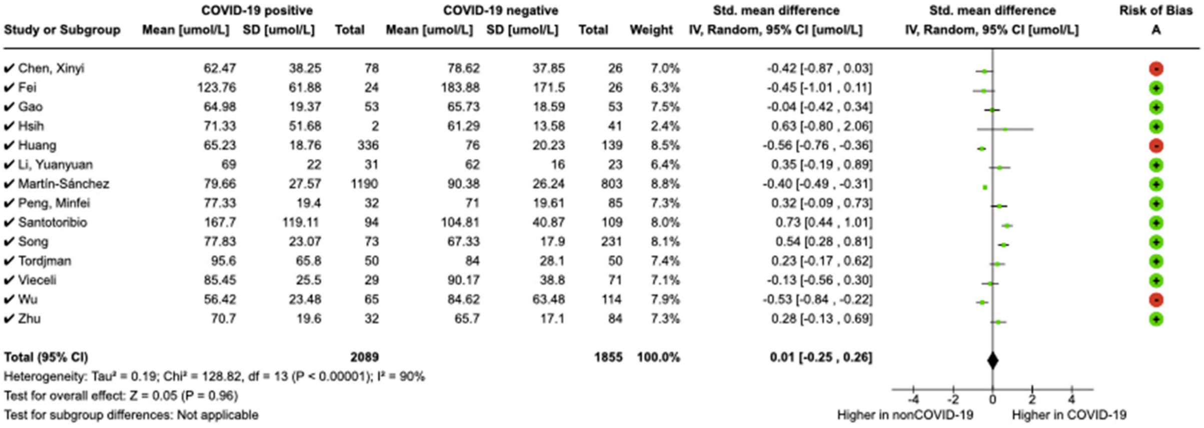

Sensitivity analysis – low risk of bias studies

Study or Subgroup	COVID-19 positive Mean [umol/L]	SD [umol/L]	Total	COVID-19 negative Mean [umol/L]	SD [umol/L]	Total	Weight	Std. mean difference IV, Random, 95% CI [umol/L]	Risk of Bias A
✖ Chen, Xinyi	62.47	38.25	78	78.62	37.85	26	0.0%	-0.42 [-0.87 , 0.03]	-
✔ Fei	123.76	61.88	24	183.88	171.5	26	8.4%	-0.45 [-1.01 , 0.11]	+
✔ Gao	64.98	19.37	53	65.73	18.59	53	9.7%	-0.04 [-0.42 , 0.34]	+
✔ Hsih	71.33	51.68	2	61.29	13.58	41	3.6%	0.63 [-0.80 , 2.06]	+
✖ Huang	65.23	18.76	336	76	20.23	139	0.0%	-0.56 [-0.76 , -0.36]	-
✔ Li, Yuanyuan	69	22	31	62	16	23	8.5%	0.35 [-0.19 , 0.89]	+
✔ Martín-Sánchez	79.66	27.57	1190	90.38	26.24	803	11.1%	-0.40 [-0.49 , -0.31]	+
✔ Peng, Minfei	77.33	19.4	32	71	19.61	85	9.5%	0.32 [-0.09 , 0.73]	+
✔ Santotoribio	167.7	119.11	94	104.81	40.87	109	10.3%	0.73 [0.44 , 1.01]	+
✔ Song	77.83	23.07	73	67.33	17.9	231	10.4%	0.54 [0.28 , 0.81]	+
✔ Tordjman	95.6	65.8	50	84	28.1	50	9.6%	0.23 [-0.17 , 0.62]	+
✔ Vieceli	85.45	25.5	29	90.17	38.8	71	9.4%	-0.13 [-0.56 , 0.30]	+
✖ Wu	56.42	23.48	65	84.62	63.48	114	0.0%	-0.53 [-0.84 , -0.22]	-
✔ Zhu	70.7	19.6	32	65.7	17.1	84	9.5%	0.28 [-0.13 , 0.69]	+
**Total (95% CI)**			**1610**			**1576**	**100.0%**	**0.16 [-0.16 , 0.49]**	

Heterogeneity: Tau² = 0.25; Chi² = 109.51, df = 10 (P < 0.00001); I² = 91%
Test for overall effect: Z = 0.98 (P = 0.32)
Test for subgroup differences: Not applicable

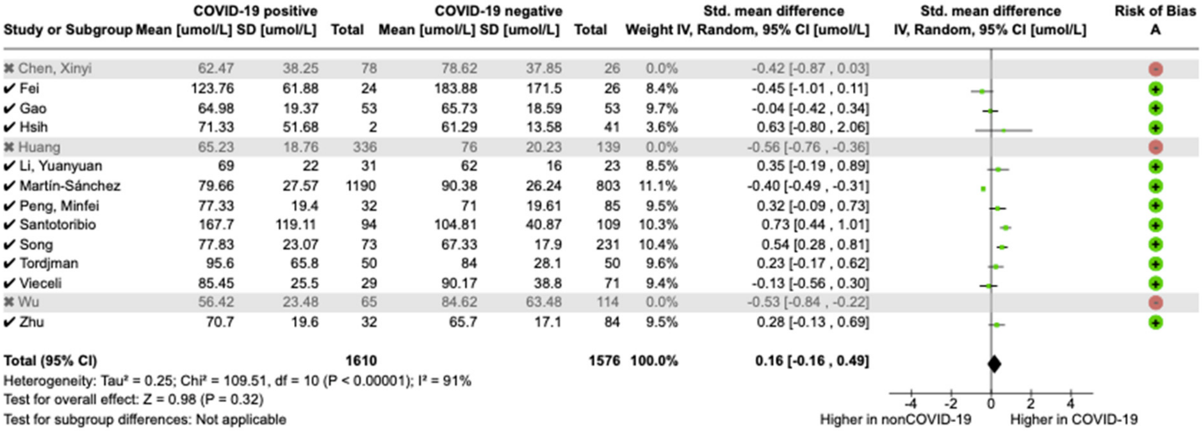

Publication bias - funnel plot

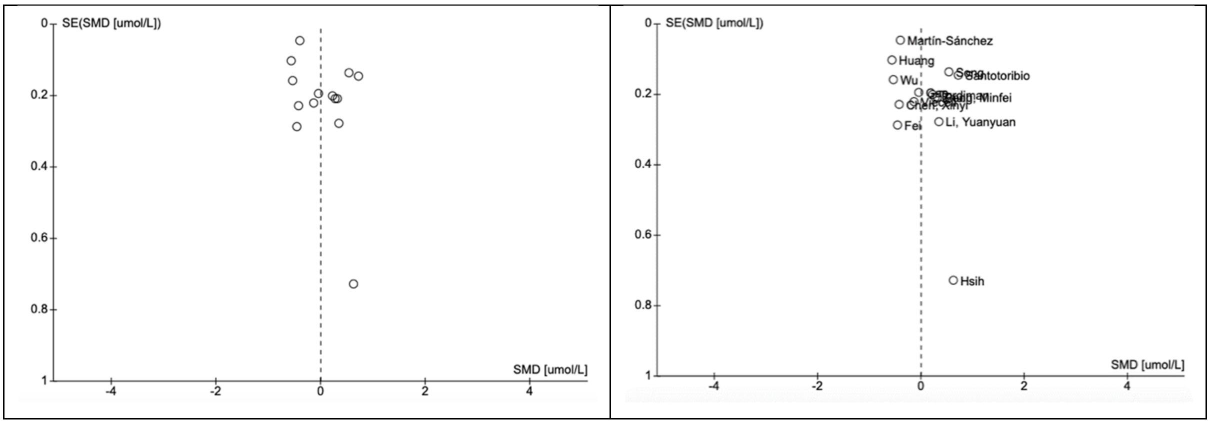

Supplement: Supplementary file 1 [file viruses-13-00803-s001.zip › Table S5 Statistical analysis.pdf]
