# Supplementary material for: Utility of Routine Laboratory Biomarkers to Detect COVID-19: A Systematic Review and Meta-Analysis
Source: Viruses. 2021 Apr 30;13(5):803. doi: 10.3390/v13050803 (PMC8147047; doi:10.3390/v13050803)
Supplement: Supplementary file 1 [file viruses-13-00803-s001.zip › Table S6 Meta-analysis.pdf]

Table S6: Meta-analysis of laboratory findings between COVID-19 and Non-COVID-19 patients, outliers not included.

Parameters	N_studies [max = 38]	Normal	Units of measurement	COVID-19 positive	COVID-19 negative	N_patients	%_positive	Std. mean difference IV (random effect model) [95% CI]	Effect size	Heterogeneity		Test for overall effect (p)	% low risk of bias
		Ranges		Total	Total					Tau²	I² (%)		
**White cell count**	33	4.0-11.0	10^9/L	4299	5483	9782	43.90%	-0.68 [-0.98, -0.38]	Large	0.72	97.00%	< 0.001	66.70%
**Neutrophil count**	28	2.0-7.0	10^9/L	4374	4667	9041	48.40%	-0.49 [-0.67, -0.31]	Medium	0.2	92.00%	< 0.001	71.40%
**Lymphocyte count**	31	1.5-4.0	10^9/L	4510	4909	9419	47.90%	-0.47 [-0.63, -0.31]	Medium	0.16	90.00%	< 0.001	64.50%
**Eosinophil count**	12	0.04-0.4	10^9/L	1520	2209	3729	40.80%	-0.85 [-1.3, -0.4]	Large	0.6	97.00%	< 0.001	58.30%
**Platelet count**	24	150-450	10^9/L	3611	4360	7971	45.30%	-0.47 [-0.66, -0.28]	Medium	0.19	92.00%	< 0.001	75.00%
**Lactate dehydrogenase**	14	135-225	U/L	1424	1321	2745	51.90%	0.42 [0.02, 0.83]	Medium	0.53	95.00%	0.04	71.40%
**Aspartate aminotransferase**	13	0-40	U/L	1421	1443	2864	49.60%	0.45 [0.26, 0.64]	Medium	0.09	79.00%	< 0.001	61.50%
**Alanine aminotransferase**	16	0-40	U/L	1493	1625	3118	47.90%	0.49 [0.25, 0.72]	Medium	0.18	87.00%	< 0.001	68.80%
**Total bilirubin**	9	<21	umol/L	2198	1662	3860	56.90%	-0.18 [-0.46, 0.11]	Small	0.15	92.00%	0.23	66.70%
**Albumin**	9	35-50	g/L	1070	998	2068	51.70%	-0.29 [-0.59, 0.01]	Medium	0.18	89.00%	0.06	55.60%
**C-Reactive Protein**	27	<5	mg/L	3866	3627	7493	51.50%	0.11 [-0.11, 0.33]	Small	0.29	94.00%	0.33	74.00%
**Procalcitonin**	9	<0.05	ng/mL	1877	1554	3431	54.70%	-0.28 [-1.03, 0.48]	Medium	1.3	99.00%	0.48	75.00%
**Creatinine**	12	45-104	umol/L	1971	1720	3691	53.40%	-0.04 [-0.28, 0.21]	Small	0.14	87.00%	0.77	57.10%
**Blood urea nitrogen**	5	2.5-7.8	mmol/L	581	552	1133	51.30%	0.15 [-0.57, 0.86]	Small	0.63	96.00%	0.69	40.00%

**D-Dimer FEU**	6	<0.1	mg/L	1674	1187	2861	58.50%	0.07 [-0.37, 0.51]	Small	0.27	94.00%	0.76	66.70%

U/L = units per litre, CI = confidence interval, IV = weighted mean difference. *Normal ranges from Newcastle Laboratories.*
