# Supplementary material for: Utility of Routine Laboratory Biomarkers to Detect COVID-19: A Systematic Review and Meta-Analysis
Source: Viruses. 2021 Apr 30;13(5):803. doi: 10.3390/v13050803 (PMC8147047; doi:10.3390/v13050803)
Supplement: Supplementary file 1 [file viruses-13-00803-s001.zip › Table S7 Sensitivity analysis Low risk of bias.pdf]

Table S7: Sensitivity analysis of laboratory findings between COVID-19 and Non-COVID-19 patients, high-risk of bias studies excluded, outliers excluded.

Parameters	N_studies [max = 26]	Normal	Units of measurement	COVID-19 positive	COVID-19 negative	Std. mean difference IV (random effect model) [95% CI]	Effect size	Heterogeneity		Test for overall effect (p)
		Ranges		Total	Total			Tau^2	I^2 (%)	
**White cell count**	22	4.0-11.0	10^9/L	2930	3287	-0.5 [-0.78, -0.23]	Medium	0.37	95%	< 0.001
**Neutrophil count**	20	2.0-7.0	10^9/L	3169	2773	-0.51 [-0.72, -0.3]	Large	0.19	91%	< 0.001
**Lymphocyte count**	20	1.5-4.0	10^9/L	3164	2846	-0.4 [-0.57, -0.23]	Medium	0.11	86%	< 0.001
**Eosinophil count**	8	0.04-0.4	10^9/L	605	573	-0.42 [-0.71, -0.13]	Medium	0.13	81%	0.004
**Platelet count**	18	150-450	10^9/L	2544	2529	-0.39 [-0.5, -0.28]	Medium	0.03	61%	< 0.001
**Lactate dehydrogenase**	12	135-225	U/L	2406	1865	0.44 [0.05, 0.84]	Medium	0.42	96%	0.03
**Aspartate aminotransferase**	10	0-40	U/L	971	1154	0.64 [0.22, 0.68]	Large	0.08	78%	< 0.001
**Alanine aminotransferase**	12	0-40	U/L	1019	1310	0.48 [0.25, 0.7]	Medium	0.11	81%	< 0.001
**Total bilirubin**	7	<21	umol/L	1784	1497	-0.08 [-0.42, 0.26]	Small	0.16	92%	0.64
**Albumin**	5	35-50	g/L	552	570	-0.14 [-0.5, 0.23]	Small	0.13	81%	0.46
**C-Reactive Protein**	21	<5	mg/L	2824	2697	0.17 [-0.07, 0.41]	Small	0.25	92%	0.26
**Procalcitonin**	7	<0.05	ng/mL	1463	1389	-0.18 [-1.12, 0.77]	Small	1.58	99%	0.71
**Creatinine**	9	45-104	umol/L	1492	1441	0.15 [-0.18, 0.48]	Small	0.2	89%	0.36
**Blood urea nitrogen**	2	2.5-7.8	mmol/L	97	257	0.30 [-0.61, 1.21]	Medium	0.38	89%	0.52

**D-Dimer FEU**	7	<0.1	mg/L	1456	1183	0.17 [-0.27, 0.61]	Small	0.31	93%	0.44

U/L = units per litre, CI = confidence interval, IV = weighted mean difference.
