# Supplementary material for: Utility of Routine Laboratory Biomarkers to Detect COVID-19: A Systematic Review and Meta-Analysis
Source: Viruses. 2021 Apr 30;13(5):803. doi: 10.3390/v13050803 (PMC8147047; doi:10.3390/v13050803)
Supplement: Supplementary file 1 [file viruses-13-00803-s001.zip › Table S8 Sensitivity analysis Published studies.pdf]

Table S8: Sensitivity analysis of laboratory findings between COVID-19 and Non-COVID-19 patients, pre-prints excluded, outliers excluded.

Parameters	N_studies [max = 30]	Normal	Units of measurement	COVID-19 positive	COVID-19 negative	Std. mean difference IV (random effect model) [95% CI]	Effect size	Heterogeneity		Test for overall effect (p)
		Ranges		Total	Total			Tau^2	I^2 (%)	
**White cell count**	25	4.0-11.0	10^9/L	3484	4259	-0.80 [-1.14, -0.45]	Large	0.69	97%	< 0.001
**Neutrophil count**	20	2.0-7.0	10^9/L	3925	3850	-0.54 [-0.76, -0.33]	Large	0.19	93%	< 0.001
**Lymphocyte count**	24	1.5-4.0	10^9/L	4077	4094	-0.51 [-0.69, -0.33]	Large	0.15	90%	< 0.001
**Eosinophil count**	8	0.04-0.4	10^9/L	1288	1858	-1.03 [-1.63, -0.43]	Large	0.72	98%	< 0.001
**Platelet count**	16	150-450	10^9/L	2796	3162	-0.48 [-0.76, -0.20]	Medium	0.29	95%	< 0.001
**Lactate dehydrogenase**	12	135-225	U/L	1286	976	0.33 [-0.15, 0.80]	Medium	0.63	95%	< 0.001
**Aspartate aminotransferase**	10	0-40	U/L	901	715	0.38 [0.16, 0.59]	Medium	0.07	70%	< 0.001
**Alanine aminotransferase**	12	0-40	U/L	941	812	0.39 [0.10, 0.69]	Medium	0.21	86%	< 0.001
**Total bilirubin**	7	<21	umol/L	1784	1194	-0.27 [-0.51, -0.03]	Medium	0.07	78%	< 0.001
**Albumin**	6	35-50	g/L	591	416	-0.21 [-0.65, 0.23]	Medium	0.26	89%	< 0.001
**C-Reactive Protein**	21	<5	mg/L	3454	2879	0.08 [-0.17, 0.33]	Small	0.28	94%	0.54
**Procalcitonin**	7	<0.05	ng/mL	1754	1161	-0.17 [-1.02, 0.67]	Small	1.25	98%	0.69
**Creatinine**	9	45-104	umol/L	1801	1290	-0.12 [-0.34, 0.11]	Small	0.07	77%	< 0.001
**Blood urea nitrogen**	3	2.5-7.8	mmol/L	484	295	0.05 [-1.09, 1.19]	Small	0.98	98%	< 0.001

**D-Dimer FEU**	7	<0.1	mg/L	1760	1168	0.22 [-0.17, 0.57]	Medium	0.19	91%	< 0.001

U/L = units per litre, CI = confidence interval, IV = weighted mean difference.
